# Ribosomal and Immune Transcripts Associate with Relapse in Acquired ADAMTS13-Deficient Thrombotic Thrombocytopenic Purpura

**DOI:** 10.1371/journal.pone.0117614

**Published:** 2015-02-11

**Authors:** Contessa E. Edgar, Deirdra R. Terrell, Sara K. Vesely, Jonathan D. Wren, Igor M. Dozmorov, Timothy B. Niewold, Michael Brown, Fang Zhou, Mark Barton Frank, Joan T. Merrill, Johanna A. Kremer Hovinga, Bernhard Lämmle, Judith A. James, James N. George, A. Darise Farris

**Affiliations:** 1 Arthritis and Clinical Immunology Program, Oklahoma Medical Research Foundation (OMRF), Oklahoma City, Oklahoma, United States of America; 2 Department of Biostatistics & Epidemiology, University of Oklahoma Health Sciences Center (OUHSC), Oklahoma City, Oklahoma, United States of America; 3 Division of Rheumatology and Department of Immunology, Mayo Clinic, Rochester, Minnesota, United States of America; 4 Clinical Pharmacology Program, OMRF, Oklahoma City, Oklahoma, United States of America; 5 Department of Medicine, OUHSC, Oklahoma City, Oklahoma, United States of America; 6 Department of Hematology & Central Hematology Laboratory, Inselspital, Bern University Hospital & University of Bern, Bern, Switzerland; National Cerebral and Cardiovascular Center, JAPAN

## Abstract

Approximately 40% of patients who survive acute episodes of thrombotic thrombocytopenic purpura (TTP) associated with severe acquired ADAMTS13 deficiency experience one or more relapses. Risk factors for relapse other than severe ADAMTS13 deficiency and ADAMTS13 autoantibodies are unknown. ADAMTS13 autoantibodies, TTP episodes following infection or type I interferon treatment and reported ensuing systemic lupus erythematosus in some patients suggest immune dysregulation. This cross-sectional study asked whether autoantibodies against RNA-binding proteins or peripheral blood gene expression profiles measured during remission are associated with history of prior relapse in acquired ADAMTS13-deficient TTP. Peripheral blood from 38 well-characterized patients with autoimmune ADAMTS13-deficient TTP in remission was examined for autoantibodies and global gene expression. A subset of TTP patients (9 patients, 24%) exhibited a peripheral blood gene signature composed of elevated ribosomal transcripts that associated with prior relapse. A non-overlapping subset of TTP patients (9 patients, 24%) displayed a peripheral blood type I interferon gene signature that associated with autoantibodies to RNA-binding proteins but not with history of relapse. Patients who had relapsed bimodally expressed higher HLA transcript levels independently of ribosomal transcripts. Presence of any one potential risk factor (ribosomal gene signature, elevated *HLA-DRB1*, elevated *HLA-DRB5*) associated with relapse (OR = 38.4; p = 0.0002) more closely than any factor alone or all factors together. Levels of immune transcripts typical of natural killer (NK) and T lymphocytes positively correlated with ribosomal gene expression and number of prior episodes but not with time since the most recent episode. Flow cytometry confirmed elevated expression of cell surface markers encoded by these transcripts on T and/or NK cell subsets of patients who had relapsed. These data associate elevated ribosomal and immune transcripts with relapse history in acquired, ADAMTS13-deficient TTP.

## Introduction

Thrombotic thrombocytopenic purpura (TTP) associated with severe, acquired ADAMTS13 deficiency is an uncommon, acute episodic disorder with risk for relapse [1]. Persistence of severe ADAMTS13 deficiency during clinical remission of some patients [1], and delayed occurrence of TTP until adulthood in other individuals with complete genetic deficiency of ADAMTS13 activity [2] suggest that ADAMTS13 deficiency alone may be insufficient to initiate acute clinical TTP episodes. Documentation of acute episodes of TTP following infection [3], inflammation [4], and pharmacologic treatment with interferon-α, a type I interferon (IFN) [5], suggests that inflammation is a possible trigger of acute episodes in this disorder.

Since the advent of plasma exchange to treat TTP, survival of the initial episode improved from 20% to 70–79% [1,6]. Improved survival rates subsequently revealed potential for relapse [7,8]. Reported risk factors for relapse susceptibility among idiopathic TTP patients include severe deficiency of ADAMTS13 activity (<10%) at the time of an episode [1] or during remission [9]. However, only 40% of TTP patients with severely deficient ADAMTS13 will relapse [1]. This observation and the absence of a clear relationship between ADAMTS13 levels during remission and subsequent relapse [1] suggest a need for additional markers of relapse. Importantly, the biological basis of relapse in ADAMTS13-deficient TTP is unknown.

The discovery of ADAMTS13 antibody responses in patients with acquired TTP [10,11] positions this disease within the spectrum of autoimmune disorders. In a previous study of 31 ADAMTS13-deficient TTP patients, 9 had other autoimmune co-morbid conditions, including non-destructive polyarthritis, Raynaud’s phenomenon, autoimmune endocrinopathies, discoid lupus and systemic lupus erythematosus (SLE) [12]. TTP shares several characteristics with SLE, including demographic population targeted [13] and flares or episodes separated by periods of relative health. SLE can clinically appear as thrombotic microangiopathy and is a differential diagnosis for observation of thrombocytopenia and microangiopathic hemolytic anemia [14,15]. A review of case reports found 87 patients having clinical evidence for both TTP and SLE [13]. A close relationship was demonstrated between childhood-diagnosed idiopathic TTP and later partial or complete SLE diagnosis [16]. More recently, we showed a great increase in the prevalence of SLE among TTP survivors [17]. In addition, anti-nuclear autoantibodies (ANA), typical of though not specific for SLE, were detected in patients with acute and quiescent TTP [12,13].

Elevated type I IFN, promoted by immune complexes comprised of RNA-binding proteins, including Ro, La, Smith (Sm) and/or Nuclear Ribonuclear Protein (nRNP) [18], bound to anti-RNA-binding protein-specific autoantibodies, has emerged as a major driver of immune dysregulation in SLE [19]. Such RNA-containing immune complexes activate plasmacytoid dendritic cells to produce type I IFN by triggering RNA-binding Toll-like receptors following Fc receptor-mediated uptake [20–22]. Stimulation of RNA-binding Toll-like receptors in plasmacytoid dendritic cells normally promotes immune responses to viral pathogens. In SLE patients, however, elevated serum type I IFN activity and/or increased type I IFN-responsive gene expression associates with autoantibodies specific for RNA-binding proteins [23], elevated disease activity [23–26], particular genetic polymorphisms [27] and major multi-organ involvement [28].

The discovery of ANAs in patients with ADAMTS13-deficient TTP [12], increased prevalence of SLE after survival of TTP [17] and case reports of TTP episodes following interferon- therapy [5] prompted us to examine ADAMTS13-deficient TTP patients in remission for evidence of underlying ANA-driven, type I IFN-mediated inflammation and further test for possible association with relapse. We report here that a subset of TTP patients have a type I IFN peripheral blood gene signature that associates with autoantibodies to RNA-binding proteins. However, these linked features did not associate with history of TTP relapse. In contrast, a ribosomal gene signature (RGS) and select immune transcripts commonly expressed in T and natural killer (NK) lymphocytes demonstrated significant association with history of relapse in ADAMTS13-deficent TTP patients in remission.

## Patients and Methods

### Patient recruitment and blood sampling

Patients were recruited from the Oklahoma TTP Registry. TTP was defined clinically by the presence of thrombocytopenia and microangiopathic hemolytic anemia without another apparent etiology. In all patients, the diagnosis of TTP was supported by the presence of severe ADAMTS 13 deficiency (activity <10%). In all patients the TTP was acquired, not hereditary, documented by the presence of an inhibitor of ADAMTS13 activity, a presumed autoantibody, and/or the recovery of ADAMTS13 activity during remission. A relapse is defined as the recurrence of an acute episode of TTP, manifested by thrombocytopenia and microangiopathic hemolytic anemia with severe ADAMTS13 deficiency, occurring more than 30 days following occurrence of a remission. All patients were required to be episode-free for at least 2 months for inclusion in the study. The only exclusion criterion was previous diagnosis with a rheumatic autoimmune disorder. Two patients (one SLE patient and one Sjögren’s syndrome patient) were excluded. Within the Registry, 41 living patients met eligibility criteria. Blood samples were collected from 38 (93%) of 41 eligible TTP subjects during disease remission in 2009 for gene expression studies and antibody measurements. Of the 38 patients included in the study, 16 had a history of relapse. For flow cytometry studies only, blood was collected from a subset of the same patients (n = 9 relapse, n = 13 non-relapse) in 2012. None of the subjects experienced a relapse during the three year time period between blood draws. Patients received immunosuppressive therapy in addition to plasma exchange for their acute episodes. No long-term immunosuppressive therapy to prevent relapse was used. This research was approved by the University of Oklahoma Health Sciences Center and Oklahoma Medical Research Foundation institutional review boards, ensuring compliance with the Helsinki Declaration of 1975, as revised in 2008. All participants provided written informed consent.

### RNA purification and microarray procedure

Blood was collected into PAXgene Blood RNA tubes (762165, Qiagen PreAnalytiX, Hombrechtikon, Switzerland) as described by the manufacturer and stored at -80°C until processing. RNA was purified with the PAXGene Blood RNA Kit and protocol (762164, Qiagen PreAnalytiX). α and β globin RNA was reduced using the GLOBINclear Human kit (AM1980, Ambion, Austin, TX, USA). RNA quantity was measured with a Nanodrop1000 spectrophotometer, and RNA yields were 1–9 μg of RNA from 2.5 mL of whole blood. RNA quality was evaluated with an Agilent 2100 Bioanalyzer, and RNA Integrity Numbers were ≥7.6. RNA was labeled using the Illumina TotalPrep RNA Amplification Kit (IL1791, Ambion, Inc., Austin). cRNA was hybridized to Illumina WG-6 version 3 Expression BeadChips (Illumina Inc, San Diego, CA). Chips were scanned using an Illumina iSCAN scanner (Illumina, San Diego, CA, USA). Gene expression data were deposited in NCBI’s Gene Expression Omnibus [29] (GSE36418).

### Microarray gene expression analysis

Hypervariably expressed genes were identified and normalized as described previously [30]. Hierarchical clustering and heatmaps were generated with Spotfire Decision Site 9 (TIBCO, Palo Alto, CA, USA).

Differential gene expression was determined using a method that incorporates an internal-standard based approach of normalization and an associative t-test to minimize false positive determinations as previously described [31,32]. Genes exhibiting normalized expression values 20 times the standard deviation of the statistically defined background were considered expressed. Genes differentially expressed ≥1.5 fold passed the standard t-test significance level of p<0.05 and passed an associative t-test threshold to eliminate false positive determinations.

For meta-analysis of gene expression trends, 3,600 human 2-color microarrays were downloaded from NCBI’s GEO database [29] as described previously [33], and their expressions normalized so direct comparisons could be made [34]. Pearson’s correlation coefficients were calculated for every gene upregulated in relapse patients (as defined above) versus every other gene upregulated in relapse patients (as defined above) using data from these 3,600 microarrays. This analysis asks whether genes upregulated in the experiments presented herein are normally correlated with each other in other, unrelated experiments found in the public database. Differentially expressed genes above threshold were then clustered based on their Pearson’s R-values, enabling identification of recurring expression patterns (genes with prior positive correlations, in this case, the RGS) and deviations from prior expression trends (genes that may be anti-correlated, in this case, the ten gene immune subset discussed in the Results section).

### Autoantibody detection

Individual ELISAs were performed to sensitively detect serum autoantibodies to the RNA-binding proteins Ro, La, Sm and nRNP using a pre-determined threshold of positivity that was based on a value of 3 standard deviations above the mean values of a set of healthy control sera as previously described [35].

### Epithelial cell reporter assay for serum type I IFN activity

Type I IFN activity was measured in sera as described [23]. Briefly, 50% dilutions of test sera were incubated with the WISH epithelial cell line for six hours followed by quantification of WISH cell expression of the IFN-regulated genes *MX-1*, *IFIT-1* and *PKR* relative to *GAPDH* by RT-PCR. Samples in which the sum of standard deviations above a previously established standard set of healthy control sera was >2 were considered positive.

### ADAMTS13 activity

Serum ADAMTS13 activity was measured as previously described [1] using immunoblot to detect degradation of plasma-derived von Willebrand factor (vWF) substrate [36,37] and fluorescence of FRET-vWF73 substrate [38,39].

### Flow Cytometry

All samples were evaluated on the same day. Frozen PBMCs (5 × 10^5^) were thawed, washed twice with RPMI 1640 complete medium (RPMI 1640 supplemented with 10% fetal bovine serum, 2mM L-glutamine and 50 international unit (IU)/ml penicillin/streptomycin at 2.5 × 10^5^ cells/ml) with benzonase (25U/ml; Sigma) and subsequently stained with fluorescently labeled anti-human CD3 (clone HIT3a, PerCP-Cy5.5), CD56 (HCD56, APC-Cy7), CD52 (HI186, Pacific Blue), CD69 (FN50, PE-Cy5) and CD244 (Cl.7, FITC) monoclonal antibodies (Biolegend or ExBIO Praha), as well as the eFluor506 fixable viability dye (Invitrogen). Data of live-gated cells were collected using a FACS LSR II flow cytometer (Becton Dickinson) and analyzed using Flow Jo software (TreeStar).

### Statistical analysis for prevalence, associations and correlations

When evaluating categorical data associations, Fisher’s exact test was used. Odds ratios (OR) and 95% confidence intervals (CI) were calculated using the maximum likelihood estimator (MLE) where applicable. In the event of a zero cell within the contingency table, the OR and CI were calculated using the modified median unbiased estimator (MMUE) and exact bootstrap distribution, respectively, as described previously [40]. For continuous data, a Student’s *t*-test or Kolmogorov-Smirnov test was used. Correlation analyses were conducted using the Spearman test. The Mann-Whitney test was used to evaluate differences in cell surface marker expression by flow cytometry. All tests were two-tailed. Statistical calculations were performed using SAS (Version 9.2, SAS Institute, Cary, NC, USA), GraphPad Prism 6, or R (version 3.1.0).

## Results

### TTP patients in remission display ribosomal or type I IFN peripheral blood gene signatures that associate with relapse or autoantibody phenotypes

Relapse occurrence in only some ADAMTS13-deficient TTP patients [1] suggests the potential for biologic heterogeneity in the TTP patient population that could contribute to relapse risk. As a first step to identify transcriptional correlates of relapse, we elected to perform a cross-sectional study of global gene expression in the peripheral blood of ADAMTS13-deficient TTP patients in remission. To avoid detection of inflammatory signatures that are a consequence of an acute episode and associated tissue damage, all remission samples were collected after at least 2 months had elapsed since the most recent episode. Characteristics of the studied cohort of 38 patients at the time of sample collection are shown in Table 1. The median number of episodes in the relapse group was 3 (range 2–5). No significant differences between the relapse and non-relapse groups were detected in terms of age, race or sex. A slight difference in follow-up time was noted between the two groups, with 9.8 (range 3.6–26.6) and 6.8 (range 0.8–12.8) median years of follow-up for the relapse and non-relapse groups, respectively. Median times since the most recent episode did not differ between the two groups.

**Table 1:** Characteristics of TTP remission cohort^a^.

	Relapse (n = 16)	Non-Relapse (n = 22)	p-value
Age ± SD	43.9 ± 15.9	45.3 ± 12.8	0.362^b^
Race/Ethnicity % [number]			0.199^c^
Caucasian	43.8% [7]	72.7% [16]	
African American	43.8% [7]	22.7% [5]	
American Indian	0.06% [1]	0.05% [1]	
Hispanic	0.06% [1]	0% [0]	
Sex % [number]			
Female	68.8% [11]	95.5% [21]	0.065^c^
Median years follow-up [range]	9.8 [3.6–26.6]	6.8 [0.8–12.8]	0.0325^d^
Median years since last episode [range]	3.4 [0.2–13.2]	6.8 [0.8–12.8]	0.076^d^

^a^ Individuals with a history of clinically diagnosed TTP (<10% ADAMTS13 activity during a prior acute episode), in remission for at least 2 months, who fail to meet criteria for any known rheumatic disease;

^b^ t-test;

^c^ Fisher’s exact test;

^d^ Kolmogorov-Smirnov test.

**Fig 1:**
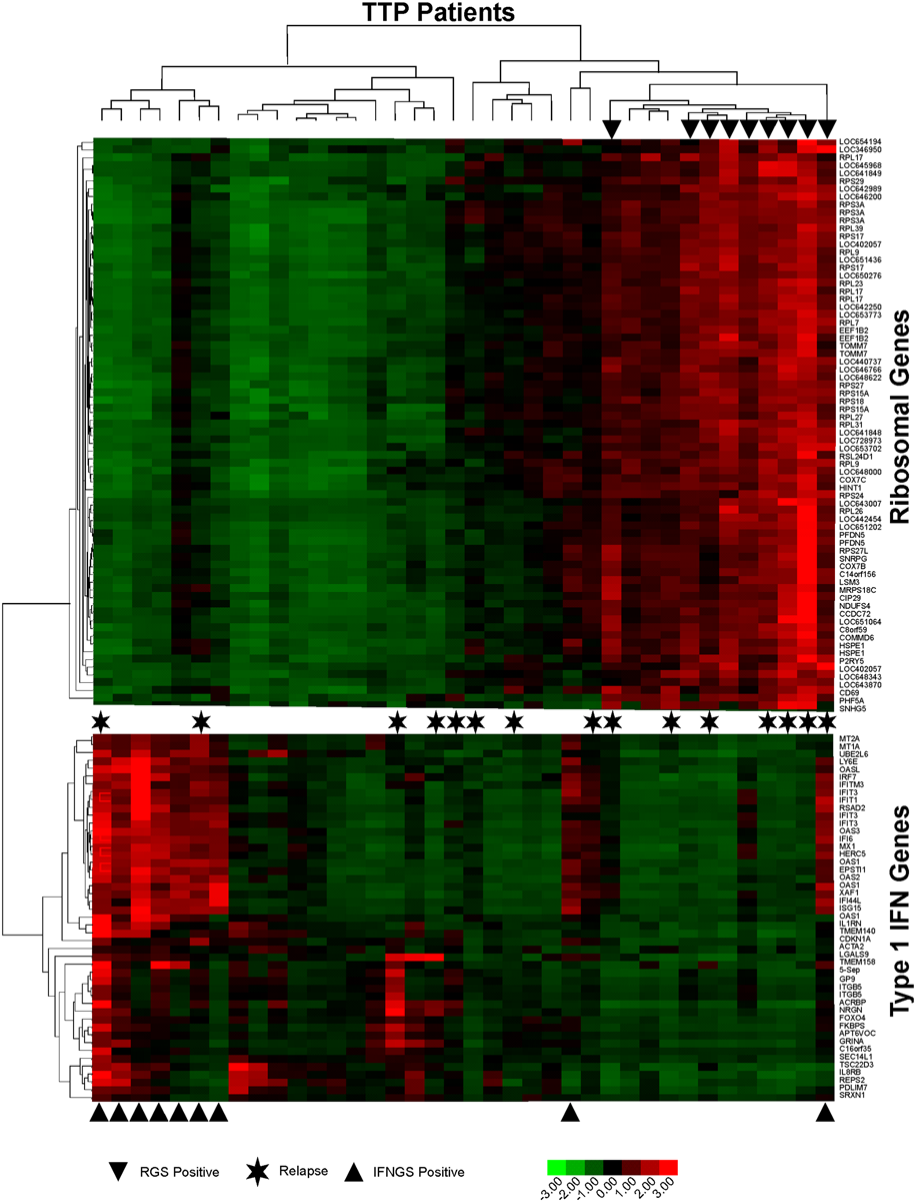
Heatmap of gene signatures present in peripheral blood of ADAMTS13-deficient TTP patients in remission. Transformed expression levels of hypervariably expressed genes from the peripheral blood of ADAMTS13-deficient TTP patients were subjected to unsupervised clustering, revealing two predominant gene signatures. Arrowheads above the charts indicate samples that were classified as positive for a ribosomal gene signature, and arrowheads below the charts indicate samples that were classified as positive for a type I IFN gene signature. Asterisks indicate patients who had experienced at least one relapse. Data were transformed to the same scale ranges, making average expression over samples of zero and standard deviation of 1.0 for each gene. Colors indicate increased expression (red) or decreased expression (green).

RNA isolated from whole peripheral blood was evaluated for global gene expression. To investigate heterogeneity of peripheral blood gene expression, hypervariably-expressed genes were identified, normalized and subjected to unsupervised hierarchical clustering. This analysis revealed two predominant clusters (Fig. 1, S1–S2 Tables). The first cluster, comprised of 47 probes (42 unique genes), contains elevated expression of 24 (57%) known type I IFN-regulated transcripts (S2 Table) and was designated the type I IFN gene signature. From this signature, the average normalized expression of the known type I IFN-induced genes was calculated, and the nine individuals with an average of ≥1.0 were classified as having a type I IFN gene signature (Fig. 1, bottom arrowheads).

Hierarchical clustering of hypervariable genes revealed a second cluster of 73 probes (57 unique transcripts; 5 putative transcripts were discontinued in the current genome assembly and therefore excluded) that encodes primarily (75%) structural ribosomal proteins or other translation machinery components. This cluster, termed the ribosomal gene signature (RGS, S1 Table), also contains genes encoding products involved in energy production and proliferation (11%), including the immune cell activation marker CD69. The nine individuals with an average normalized expression of the 73 probes of ≥1.0 were classified as having a ribosomal gene signature (Fig. 1, top arrowheads). Thus, both type I IFN and ribosomal peripheral blood gene expression signatures were identified within a group of 38 ADAMTS13-deficient TTP patients during remission, and presence of these signatures was heterogeneous within the patient population.

**Fig 2:**
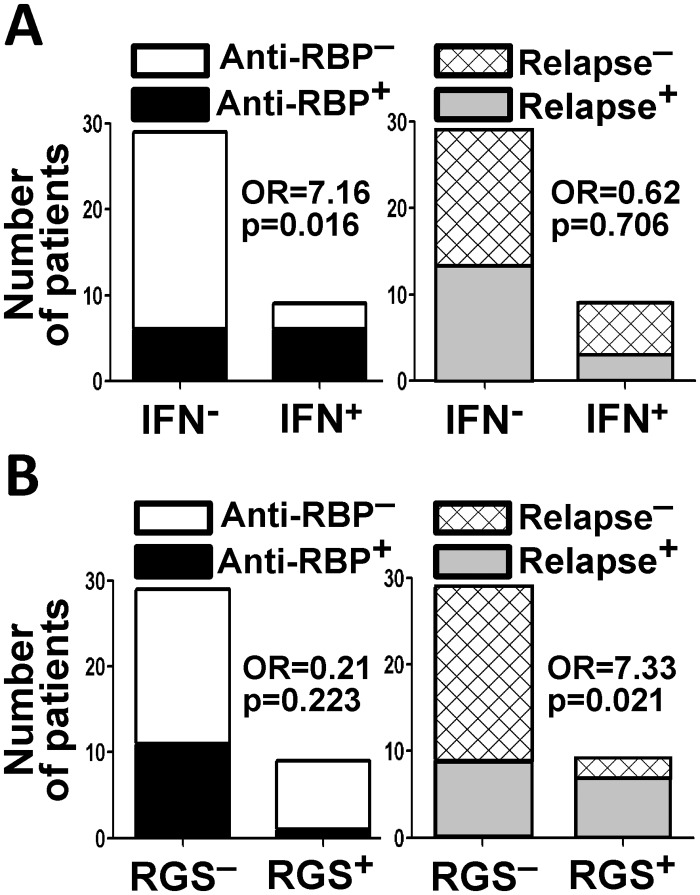
Type I IFN and ribosomal gene expression signature associations. (A) Association of the type I IFN gene signature with antibodies to RNA binding proteins (RBP) but not relapse history. (B) Association of the ribosomal gene signature (RGS) with relapse history but not antibodies to RBP. Proportions were compared using Fisher’s exact test. Odds ratios (OR) were calculated as described in Materials and Methods.

Increased type I IFN gene signatures and enhanced type I IFN activity have been associated with autoantibodies specific for RNA-binding proteins in the autoimmune disease SLE [23]. To determine if a similar association is present in ADAMTS13-deficient TTP patients without any known overlapping rheumatic diseases, the patient serum samples were tested for autoantibodies to the Ro, La, Sm and nRNP RNA-binding proteins using antigen-specific ELISAs and the data compared with presence of the type 1 IFN gene signature. Of the 38 patients tested, 12 had detectable antibodies to one or more of these RNA binding antigens, with the following pattern of reactivity: Ro only = 7 patients, Ro and nRNP = 2 patients, nRNP only = 1 patient, Sm and nRNP = 1 patient, La only = 1 patient. Positivity for the type I IFN signature associated with presence of elevated serum type I IFN activity, with 4 of 9 (44%) IFN signature positive patients having high type 1 IFN activity and 2 of 29 (7%) IFN signature negative patients having high type 1 IFN activity (p = 0.020; OR = 9.85, 95%CI 1.09–137.4, S1 Fig.). Positivity for the IFN signature also associated with presence of autoantibodies specific for RNA-binding proteins, with 6 of 9 (66%) IFN signature positive patients and 6 of 29 (10%) IFN signature negative patients producing the antibodies (p = 0.016; OR = 7.16, 95% CI 1.14–58.23; Fig. 2A left). Notably, however, the type I IFN signature did not associate with history of relapse (p = 0.706; OR = 0.62, 95% CI 0.08–3.64; Fig. 2A right).

**Fig 3:**
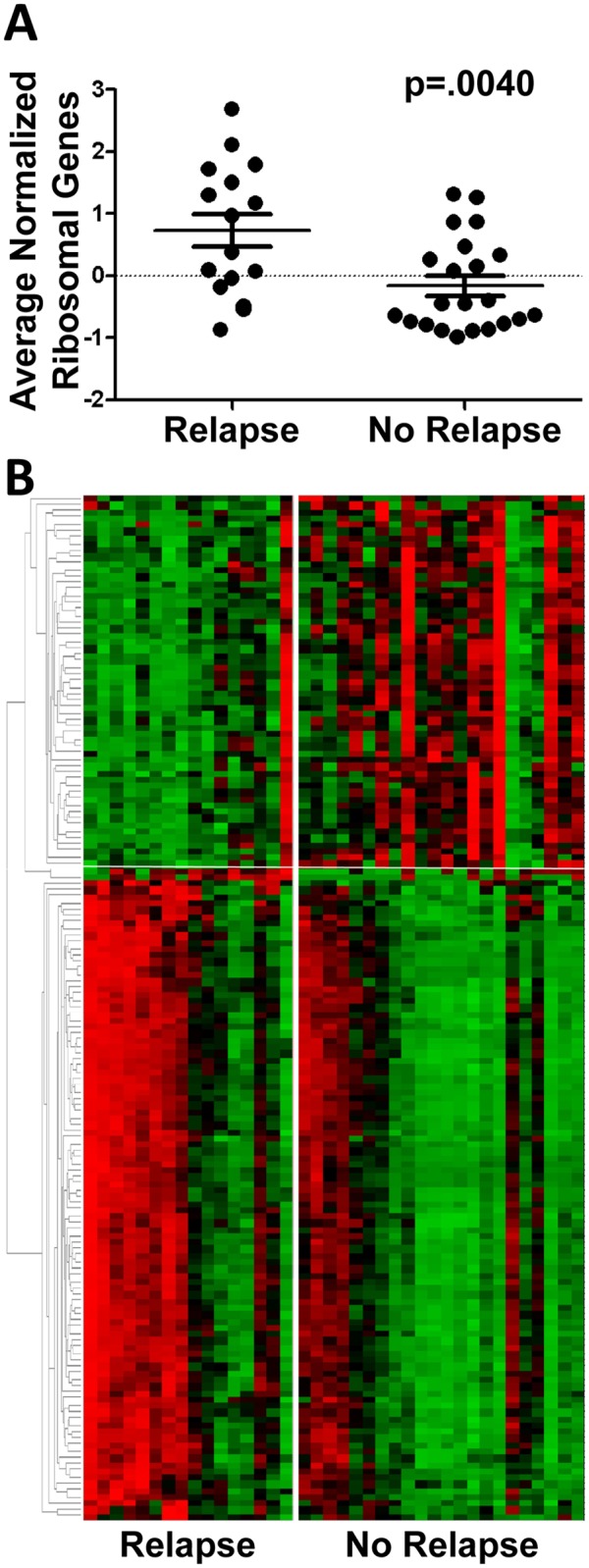
Direct comparison of gene expression of peripheral blood samples between patients with or without a history of relapse. (A) Average normalized ribosomal gene signature expression values in patients with or without a history of relapse. Error bars depict standard error of the mean. Expression values were compared using Student’s t-test. (B) Relative expression levels of genes differentially expressed between relapse and non-relapse patients presented following clustering. Colors indicate relative increased (red) or decreased (green) expression.

The patient data were then analyzed to determine whether the RGS associates with presence of autoantibodies to RNA-binding proteins or relapse history. While no association was detected between the RGS and autoantibodies to RNA-binding proteins (p = 0.223; OR = 0.21, 95%CI 0.004–1.97; Fig. 2B), the RGS significantly associated with relapse history, as the proportion of ribosomal gene signature-positive patients who had experienced one or more relapses (7 of 9, 78%) is significantly higher than the proportion of ribosomal gene signature-negative patients who had experienced one or more relapses (9 of 29, 31%; p = 0.021, OR = 7.33, 95% CI 1.11–86.14; Fig. 2B). Moreover, the average ± SEM of normalized levels of ribosomal gene expression in relapse patients (0.729±0.267) is higher than that of non-relapse patients (-0.162±0.158, p = 0.0040, Fig. 3A).

### TTP patients with a history of relapse differentially express ribosomal and immune genes

To identify relapse-associated transcripts in ADAMTS13-deficient TTP more precisely, genes differentially expressed between patients with or without a history of relapse were determined. Within the relapse group, 98 probes detected 81 unique downregulated genes (ranging from 1.5 to 1.83 fold downregulation), while 142 probes detected 121 unique upregulated genes (ranging from 1.5 to 13 fold upregulation, Fig. 3B, S3 and S4 Tables). Over 46% of transcripts downregulated in relapse patients are expressed in neutrophils, including *IL-8*, *CXCR1*, *FCGR2A*, *VNN3*, *VNN2*, *NCF1B*, *FPR2*, *NCF1* (S3 Table). Despite this observation, complete blood cell counts revealed that all peripheral blood cell subsets, including granulocytes, were within normal limits, with no detectable differences between the relapse and non-relapse groups (S2 Fig.).

As expected, a large proportion (61%) of genes upregulated in the relapse group encode structural ribosomal proteins, components of translation machinery and protein products involved in energy metabolism, thus confirming the association of a ribosomal gene signature with relapse (S4 Table). Several transcripts encoding proteins functioning in the immune system were overexpressed in the relapse group, including *HLA-DRB1* (2.0 fold), *HLA-DRB5* (3.4 fold), *IFNG* (2.3 fold), *CD69* (1.7 fold), *GZMA* (1.7 fold), *P2RY5* (1.6 fold), *KLRB1/CD161* (1.6 fold), *CD244* (1.6 fold), *CD52* (1.6 fold), *MAF* (1.6 fold) and *HOPX* (1.6 fold). A meta-analysis of expression of the upregulated genes using publically available data from 3,600 other human microarray experiments revealed that the expression levels of ribosomal genes generally correlate with one another in settings other than TTP (S3 Fig.). Specifically, a total of 63 relapse-upregulated genes mapped to probes within these arrays, and 69% (2,727/3,969) of the Pearson’s correlations were significant at p<0.01. Expression levels of 10 of the 11 relapse-elevated immune transcripts (except *CD69*) also correlated with one another in other experiments. Interestingly, however, expression of these ten immune genes is ordinarily anti-correlated with that of the ribosomal genes. That is, they tend not to be upregulated together with ribosomal genes in the other experiments analyzed, as they were in relapsing TTP patients, suggesting their aberrant regulation in TTP.

**Fig 4:**
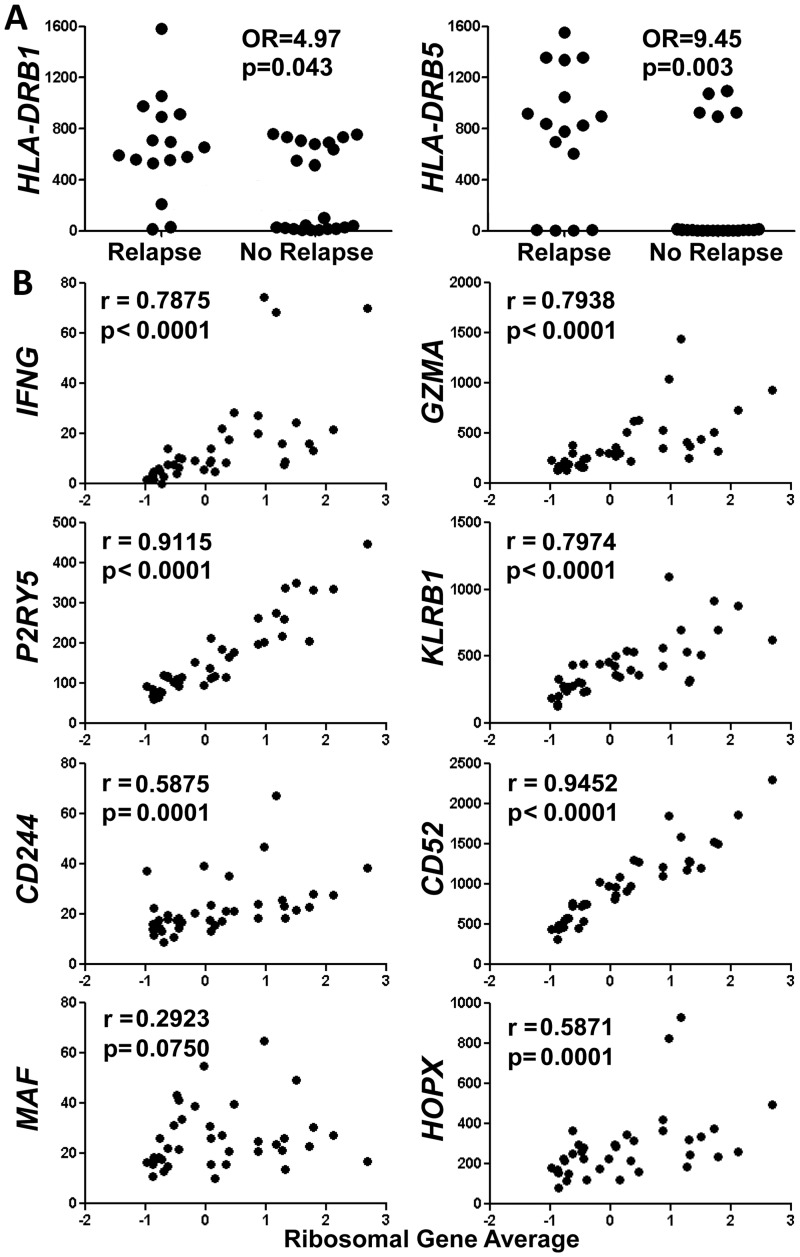
Relationship of relapse-over-expressed immune genes to the ribosomal gene signature. (A) Association of increased *HLA-DRB1* and *HLA-DRB5* expression with history of relapse. Proportions were compared using Fisher’s exact test. Y-axes are in normalized units. Expression levels >400 normalized units were considered high. (B) Positive correlation of the patient ribosomal gene signature with expression of seven of eight immune genes over-expressed in relapse patients. Spearman r values are shown. Y-axes are in normalized units.

Within these ten immune genes, *HLA-DRB1* and *HLA-DRB5* expression was bimodal, with average ± SEM in *DRB1* high (n = 23) and low (n = 15) groups equal to 740.6±48.6 and 38.1±13.5, respectively, and average ± SEM in *DRB5* high (n = 17) and low (n = 21) groups equal to 1007.0±63.0 and 4.2±0.8, respectively. High expression of these HLA-DR genes associated with relapse (OR = 4.97, p = 0.043 for DRB1 and OR = 9.45, p = 0.003 for DRB5; Fig. 4A and Table 2). Notably, all individuals with high expression of *DRB5* also exhibited high expression of *DRB1*. High expression of these human leukocyte antigen (HLA) genes was not associated with presence of the ribosomal gene signature (S4 Fig.), indicating that association of *HLA-DRB1* and-*DRB5* expression with relapse is ribosomal gene signature-independent. Since the RGS and high expression of *DRB1* and *DRB5* independently associated with relapse, we asked whether presence of a combination of these factors would more strongly associate with relapse. Presence of all three factors was significantly associated with relapse (Table 2). However, the presence of any one of the factors was more strongly associated with relapse (OR = 38.5, p = .002 for “RGS^+^ or DRB1^hi^ or DRB5^hi^”, Table 2) than having all of the factors together or any one factor alone.

**Table 2:** Association of RGS, HLA DR gene expression or combination thereof with relapse in ADAMTS13-deficient TTP.

Model	OR ^a^	Lower 95% CI	Upper 95% CI	p-Value ^c^
RGS^+^	7.78	1.53	59.71	0.021
DRB1^hi^	4.97	0.97	34.97	0.043
DRB5^hi^	9.45	1.86	61.15	0.003
RGS^+^ & DRB1^hi^ & DRB5^hi^	21.08 ^a^	2.05 ^b^	43.84 ^b^	0.025
RGS^+^ or DRB1^hi^ or DRB5^hi^	38.35 ^a^	4.98 ^b^	85.22 ^b^	0.002

^a^ Odds ratios (OR) estimated by modified median unbiased estimation (MMUE) due to zero cells;

^b^ Confidence intervals (CI) estimated by exact, bootstrapped MMUE;

^c^ Fisher’s exact test, 2-tailed.

**Fig 5:**
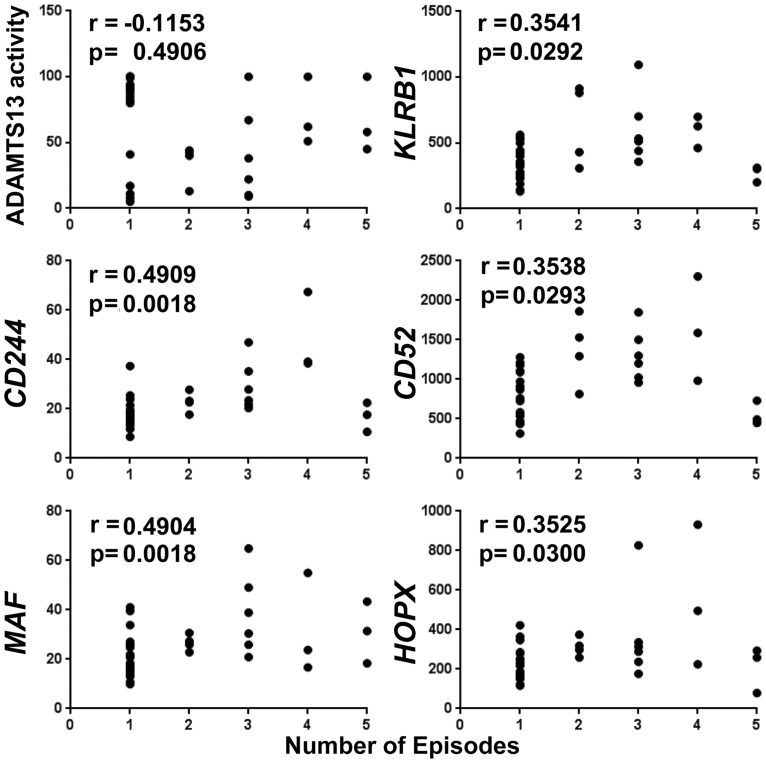
Relationship between peripheral blood markers and number of episodes. Expression of five immune transcripts overexpressed in patients with history of relapse correlates with number of past TTP episodes. Serum ADAMTS13 activity does not correlate with number of past episodes (upper left panel). Y-axis is percent of full activity for ADAMTS13 as measured by FRET assay (upper left panel). Y-axis is gene expression in normalized units for the five immune genes (all other panels). Spearman R values are shown.

In contrast to *HLA-DRB1* and-*DRB5* transcripts, expression of *IFNG* (r = 0.7875), *GZMA* (r = 0.7938), *P2RY5* (r = 0.9115), *KLRB1/CD161* (0.7974), *CD244* (r = 0.5875), *CD52* (r = 0.9452) and *HOPX* (r = 0.5871) (but not *MAF*) demonstrated significantly high positive correlation with average ribosomal gene expression (Fig. 4B). *CD69* also positively correlated with average ribosomal gene expression (r = 0.9048, S5 Fig.). Although no correlation was observed between plasma ADAMTS13 activity levels in these remission samples and the number of acute TTP episodes a patient had previously experienced, positive correlations were found between expression of five of the immune genes (*KLRB1/CD161* (r = 0.3541), *CD244* (r = 0.4909), *CD52* (r = 0.3538), *MAF* (r = 0.4904), *HOPX* (r = 0.3525) and number of episodes (Fig. 5). Furthermore, neither levels of the ten immune transcripts nor average ribosomal gene expression nor plasma ADAMTS13 activity levels were correlated with time elapsed since the most recent episode (S6 Fig.), indicating that gene expression levels of these transcriptional correlates of relapse are unlikely to reflect inflammation caused by the most recent prior acute episode.

Plasma ADAMTS13 activity levels in the remission samples exhibited modest negative correlations with transcript levels of *MAF* (r = -0.372 [95% CI-0.624 to-0.050], p = 0.021) and *HOPX* (r = -0.367 [95% CI-0.621 to-0.044], p = 0.023), genes that are both expressed in T helper cells (S5 Table). No correlation of remission ADAMTS13 activity levels were observed with transcript levels of *IFNG*, *GZMA*, *P2RY5*, *KLRB1/CD161*, *CD244* or *CD52*, or with averaged normalized values of ribosomal gene expression (S5 Table). No association of remission ADAMTS13 activity levels with relapse status, high *DRB1* expression, high *DRB5* expression, presence of RGS or presence of IFN gene signatures was observed (S6 Table).

### Altered expression of cell surface markers on NK and T lymphocytes in TTP patients with a history of relapse

**Fig 6:**
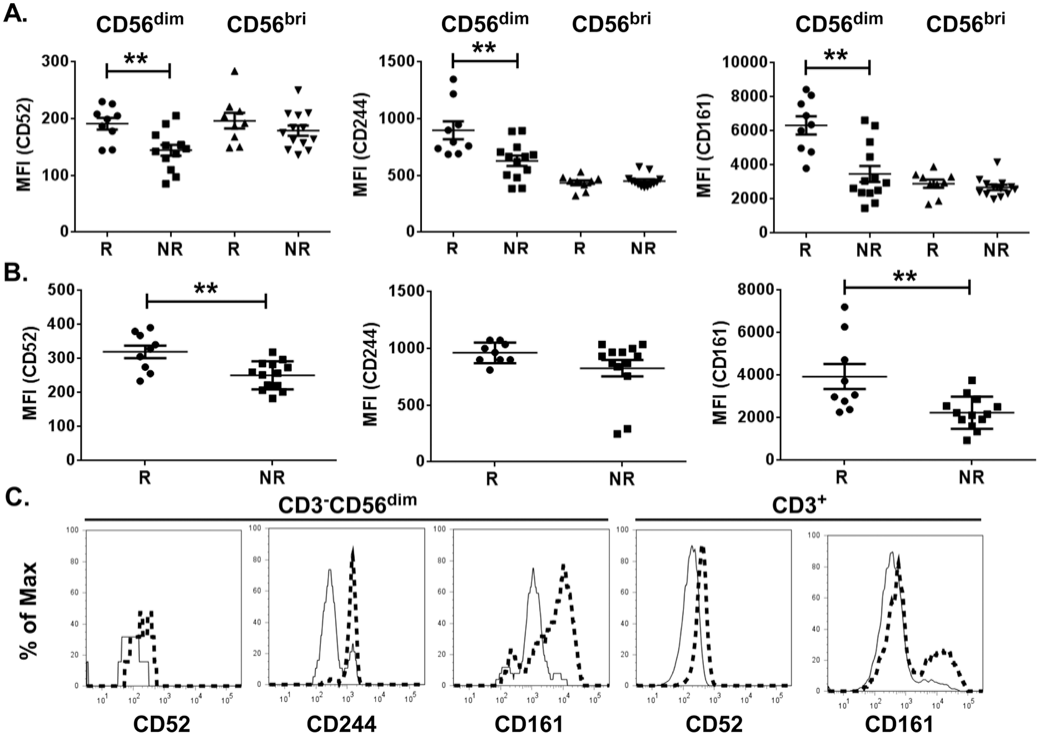
Expression of CD52, CD244 and CD161 protein on the cell surface of CD3^-^CD56^dim^ NK and CD3^+^ T cells isolated from relapsed ADAMTS13-deficient TTP patients in remission. Mean fluorescence intensity (MFI) of CD52, CD244 and CD161 on CD3^-^CD56^dim^ and CD3^-^CD56^bright^ NK cells (A) and on CD3^+^ T cells (B) derived from relapsed (R) or non-relapsed (NR) TTP patients. Representative histograms of fluorescence intensity of markers exhibiting significant differences between relapse (thick, dashed lines) and non-relapsed (thin, solid lines) patients are shown in C. (relapse n = 9, non-relapse n = 13, *Mann-Whitney test*; **p<0.01).

Of the relapse-overexpressed immune transcripts, at least nine are known to be expressed in NK or T lymphocytes. To investigate whether proteins encoded by these transcripts are elevated at the protein level, cell surface expression levels of CD52, CD161 and CD244, were measured on the major cytolytic (CD3^-^CD56^dim^) and minor regulatory (CD3^-^CD56^bright^) NK cell subsets detectable in human blood [41] and on CD3^+^ T lymphocytes from 22 of the patients (n = 9 relapse and n = 13 non-relapse). Compared to those from patients who had experienced only a single acute episode, CD56^dim^ but not CD56^bright^ NK cells from patients who had relapsed displayed elevated cell surface expression of CD52 (relapse: 191±10, non-relapse: 144±9 mean fluorescence intensity units ± SEM), CD161 (relapse: 6329±529, non-relapse: 3475±467) and CD244 (relapse: 899±79, non-relapse: 630±46) (Fig. 6). CD3^+^ T lymphocytes from relapsed patients expressed higher levels of CD52 (320±19) and CD161 (3937±590) compared to those from non-relapse patients (251±41 and 2241±755 for CD52 and CD161, respectively), while CD244 expression on T cells was not different between the two groups (Fig. 6). Thus, ADAMTS13-deficient TTP patients in remission who had a history of relapse displayed altered cell surface marker expression on peripheral blood NK and T lymphocytes.

## Discussion

ADAMTS13 activity and/or autoantibody levels measured during acute TTP or remission have been identified as risk factors for relapse [1,9]. However, observation of relapse in only a fraction of patients exhibiting these markers highlights both an incomplete biologic understanding of relapse and a need for additional markers. In this study we asked whether autoantibody-related type I IFN inflammation is a feature of ADAMTS13-deficient TTP and, if so, whether this inflammation is associated with relapse in this disorder. The results show that type I IFN-mediated inflammation does indeed occur in TTP but does not associate with relapse history. Therefore, we conclude that underlying elevations in type I IFN associated with the formation of RNA-containing immune complexes do not promote relapse in TTP. However, the TTP patients exhibiting a type I IFN gene signature and autoantibodies directed to RNA binding proteins might be at increased risk for developing SLE. All patients in this study fulfill the Hematologic Disorder SLE criterion [14,15], and 42% are positive for ANA, another SLE criterion. A type I IFN gene signature has been described in patients with incomplete lupus [42], and a pediatric study suggests that severely reduced ADAMTS13 activity in TTP patients might portend the onset of SLE [16].

Evaluation of hypervariable gene expression in the TTP patient group revealed a ribosomal gene signature that was significantly more common in patients with a history of relapse. Patterns of global gene expression have proven useful for predicting prognosis and flares in anti-neutrophil cytoplasmic antibody-associated vasculitis and SLE [43] and have been extensively used to uncover disease-promoting biologic pathways in a variety of clinical settings. Although no conclusions can be drawn from the present cross-sectional study regarding whether this gene expression profile elevates risk for relapse in TTP, this finding suggests that a prospective study to evaluate this question is warranted. Elevated ribosomal transcripts may suggest MYC-dependent cell growth and proliferation [44,45]. Enhanced expression of translation machinery could alternatively reflect enhanced differentiation of cell types producing large quantities of protein, such as blood cells with high granule content.

To further evaluate the association of a ribosomal gene signature with relapse in TTP, we also performed a direct comparison of differential global gene expression between the relapsed patient group compared to the non-relapsed patient group. This analysis confirmed higher expression of multiple ribosomal and translational genes in relapsers and further pinpointed unusual co-elevated expression of several transcripts typically expressed in T and NK subsets of lymphocytes, as well as increased expression of HLA class II genes in relapsed patients. The bimodal, elevated expression of *HLA-DRB1* and *HLA-DRB5* in patients with a history of relapse may signal association of a specific HLA haplotype with relapse in ADAMTS13-deficient TTP. Elevated expression of the same *HLA-DRB1* and *-DRB5* probes from the same platform utilized in the present study reflected presence of a specific HLA haplotype in a study of multiple sclerosis [46]. Although selected HLA-DR and-DQ alleles have been reported to associate with TTP [47,48], there are no reports of selected HLA alleles associating with relapse. This is an important area for further investigation, as select HLA haplotypes may pre-dispose to chronically increased ADAMTS13 autoantibodies.

Correlation of ribosomal gene expression with transcripts typically expressed in NK cells (*KLRB1/CD161*, *CD244*, *GZMA*, *IFNG*) [41,49–51] and certain subsets of T-lymphocytes (*MAF*, *HOPX*, *GZMA*, *IFNG*, *CD52*, *KLRB1/CD161*) [51–57] is consistent with activation and proliferation of one or more subsets of these cells in TTP patients with a history of relapse. Significant negative correlations between remission plasma levels of ADAMTS13 and transcripts encoding the T helper cell transcriptional regulators Hopx [52] and Maf [57] suggest that peripheral blood T helper cells could promote relapse by chronically driving ADAMTS13 autoantibodies in some patients. The involvement of T and/or NK lymphocytes is further supported by increased cell surface expression CD161, CD244 and CD52 on the CD3^+^CD56^dim^ cytolytic subset of NK cells and increased expression of CD161 and CD52 on CD3^+^ T cells from patients who had experienced relapse. Importantly, lack of correlation of the ribosomal and immune transcriptional signatures with time since the most recent episode suggests that the observed transcriptional changes are not simply the result of lingering inflammation from the most recent episode. However, there are multiple potential confounders for the analysis of the outcome of relapse, such as recent rituximab treatment, pregnancy, surgical procedures, and other events that may decrease or increase the risk for relapse in patients with acquired, autoimmune TTP. Prospective studies combing gene expression profiling and immunophenotyping are required to determine whether the relapse-associated transcriptional signatures and altered expression of NK and T lymphocyte cell surface markers reported here signify risk or consequence of relapse in ADAMTS13-deficient TTP, and we have initiated collection of samples from newly diagnosed, ADAMTS13-deficient TTP patients for this purpose.

Possible explanations underlying these observations include ADAMTS13-independent genetic variation that increases relapse risk or some form of immunologic memory or epigenetic imprint left on T and NK lymphocytes by acute TTP episodes or other unknown environmental exposures. Interestingly, NK cells [58] and cytotoxic T lymphocytes [59], as well as GZMA [60] and IFN- [61], two of their common products, can all cause endothelial damage, which has been suggested to be a likely inciting event for TTP [62]. We speculate that microthrombus-supporting endothelial damage in TTP could be promoted by ADAMTS13 autoantibody-dependent cellular cytotoxicity, in which ADAMT13 autoantibodies bound to ADAMTS13 at the endothelial surface [63] are engaged by Fc receptors on cytotoxic NK cells or cytotoxic T lymphocytes. In addition to being a mediator of cytotoxicity, Granzyme A has also been shown to stimulate pro-inflammatory IL-1, TNF and IL-6, secretion from macropahges [64]. Elevated levels of these cytokines have been observed in acute TTP [65], and acute TTP plasma has been shown to activate macrophages [66].

This study identifies an RBP autoantibody-associated type I IFN gene expression signature in a subset of TTP patients in remission that does not associate with relapse. Moreover, this cross-sectional study further reports a peripheral blood RGS, elevated expression of *HLA-DRB1* and-*DRB5* transcripts, and elevated expression of a discrete set of NK and T lymphocyte expressed immune genes as the first transcriptional correlates of relapse in ADAMTS13-deficient TTP. These markers warrant further investigation as potential predictors of relapse in this rare but deadly disorder.

## Supporting Information

S1 Table Genes within the RGS. Gene order matches clustering order in Fig. 1.
(DOCX)

S2 Table Genes within the type 1 IFN gene signature. Gene order matches clustering in Fig. 1.
(DOCX)

S3 Table Genes downregulated in patients with a history of relapse compared to patients without a history of relapse. *Average expression (AVG) and standard deviation (SD) in normalized units. P-values are from the associative t-test. Functional annotations are taken from the US National Center for Biotechnology Information (NCBI) database and the literature.
(DOCX)

S4 Table Genes upregulated in patients with a history of relapse compared to patients without a history of relapse. * Average expression (AVG) and standard deviation (SD) in normalized units. P-values are from the associative t-test. Functional annotations are taken from the US National Center for Biotechnology Information (NCBI) database and the literature.
(DOCX)

S5 Table Correlation of remission plasma levels of ADAMTS13 activity with peripheral blood transcripts. (DOCX)

S6 Table Remission plasma levels of ADAMTS13 activity stratified by relapse status, RGS, IFN GS and *HLA-DR* transcript levels. (DOCX)

S1 Fig Association of the type 1 IFN gene signature (IFN GS) with serum type I IFN activity. Proportions were compared using Fisher’s exact test. Odds ratios (OR) were calculated as described in Materials and Methods.
(DOCX)

S2 Fig Peripheral blood cell counts reveal no significant differences in subpopulation cell numbers between ADAMTS13 deficient TTP patients with history of relapse (R) or no history of relapse (NR). RBC = red blood cells, PLT = platelets, WBC = white blood cells, GRAN = granulocytes, LYMPH = lymphocytes, LYMPH = lymphocytes, MONO = monocytes, EOS = eosinophils, BASOS = basophils.
(DOCX)

S3 Fig TTP-upregulated ribosomal and immune genes do not correlate with each other in meta-analysis of gene expression data unrelated to TTP. Heatmap showing correlations among 63 selected genes in combined data from 3,600 non-TTP microarray datasets. Genes were selected based on their upregulation in the TTP relapse group in the present study. Red = positive Pearson’s correlation coefficient; Green = negative Pearson’s correleation coefficient. The self-self correlations (values = 1.0) can be seen on the diagonal as a reference for the relative intensities. Two groups of genes generally correlated in their expression are observed: ribosome-related genes (bottom right) and immune-related genes (top left). These two groups of genes—all up-regulated in these experiments—are not normally positively correlated. In contrast, these two groups of genes are positively correlated in TTP patients in remission.
(DOCX)

S4 Fig Lack of association between RGS and *HLA-DRB1* and *HLA-DRB5* expression. Proportions were compared using Fisher’s exact test. Odds ratios (OR) were calculated using the maximum likelihood estimator (MLE). Y-axis is in normalized units. Expression levels >400 normalized units were considered high.
(DOCX)

S5 Fig Correlation between normalized expression of CD69 and average normalized expression of ribosomal genes. R value is Spearman correlation.
(DOCX)

S6 Fig Lack of correlation between time since last episode and expression levels of immune genes, ADAMTS13 activity levels or RGS. For the immune genes, the Y-axis is in normalized units. ADAMTS13 activity is expressed as a percentage of maximum. Ribosomal Gene Average is expressed as average normalized expression. R values are Spearman correlations.
(DOCX)
