# Supplementary material for: Ribosomal and Immune Transcripts Associate with Relapse in Acquired ADAMTS13-Deficient Thrombotic Thrombocytopenic Purpura
Source: PLoS One. 2015 Feb 11;10(2):e0117614. doi: 10.1371/journal.pone.0117614 (PMC4324966; doi:10.1371/journal.pone.0117614)
Supplement: S1 Table — Gene order matches clustering order in Fig. 1. (DOCX) [file pone.0117614.s001.docx]

**Table S1.**

| **Symbol** | **Entrez ID** | **Accession** | **Function** | | | |  | **Symbol** | **Entrez ID** | **Accession** | **Function** | | | |
| --- | --- | --- | --- | --- | --- | --- | --- | --- | --- | --- | --- | --- | --- | --- |
| LOC654194 | 654194 | LOC654194 (d) |  |  |  |  |  | RSL24D1 | 51187 | NM_016304.2 | T | R |  |  |
| RPL37P6 | 346950 | LOC346950 (p) | T | R |  |  |  | RPL9 | 6133 | NM_001024921.2 | T | R |  |  |
| RPL17 | 6139 | NM_001035006.2 | T | R |  |  |  | RPL7P23 | 648000 | LOC648000 (p) | T | R |  |  |
| RPS3AP25 | 645968 | LOC645968 (p) | T | R |  |  |  | COX7C | 1350 | NM_001867.2 |  |  | E |  |
| LOC641849 | 641849 | LOC641849 (d) |  |  |  |  |  | HINT1 | 3094 | NM_005340.5 |  |  |  |  |
| RPS29 | 6235 | NM_001030001.1 | T | R |  |  |  | RPL36AP8 | 643007 | LOC643007 (P) | T | R |  |  |
| LOC642989 | 642989 | LOC642989 (d) |  |  |  |  |  | RPL26 | 6154 | NM_000987.3 | T | R |  |  |
| LOC646200 | 646200 | LOC64200 (p) | T | R |  |  |  | RPS24 | 6229 | NM_033022.3 | T | R |  |  |
| RPS3A | 6189 | NM_001006.3 | T | R |  |  |  | UQCRBP1 | 442454 | NR_002308.1 |  |  | E |  |
| RPS3A | 6189 | NM_001006.3 | T | R |  |  |  | RPL36AP49 | 651202 | LOC651202 (p) | T | R |  |  |
| RPS3A | 6189 | NM_001006.3 | T | R |  |  |  | PFDN5 | 5204 | NM_002624.3 | T |  |  |  |
| RPL39 | 6170 | NM_001000.2 | T | R |  |  |  | PFDN5 | 5204 | NM_145897.2 | T |  |  |  |
| RPS17 | 6218 | NM_001021.3 | T | R |  |  |  | RPS27L | 51065 | NM_015920.3 | T | R |  |  |
| RPS17P16 | 402057 | LOC402057 (p) | T | R |  |  |  | SNRPG | 6637 | NM_003096.2 | T |  |  |  |
| RPL9 | 6133 | NM_001024921.2 | T | R |  |  |  | COX7B | 1349 | NM_001866.2 |  |  | E |  |
| RPL9P25 | 651436 | LOC651436 (p) | T | R |  |  |  | C14orf156 | 81892 | NM_031210.4 | T |  |  |  |
| RPS17 | 6218 | NM_001021.3 | T | R |  |  |  | LSM3 | 27258 | NM_14779635 | T |  |  |  |
| RPL7P6 | 650276 | LOC650276 (p) | T | R |  |  |  | MRPS18C | 51023 | NM_016067.2 | T | R |  |  |
| RPL23 | 9349 | NM_000978.3 | T | R |  |  |  | CIP29 | 84324 | NM_033082.3 |  |  |  | P |
| RPL17 | 6139 | NM_000985.4 | T | R |  |  |  | NDUFS4 | 4724 | NM_002495.2 |  |  | E |  |
| RPL17 | 6139 | NM_000985.4 | T | R |  |  |  | CCDC72 | 51372 | NM_015933.3 |  |  |  |  |
| RPL17L | 642250 | LOC642250 (p) | T | R |  |  |  | LOC651064 | 651064 | LOC651064 (p) |  |  |  |  |
| RPL31P17 | 653773 | LOC653773 (p) | T | R |  |  |  | C8orf59 | 401466 | NM_001099672.1 |  |  |  |  |
| RPL7 | 6129 | NM_000971.3 | T | R |  |  |  | COMMD6 | 170622 | NM_203497.2 |  |  |  |  |
| EEF1B2 | 1933 | NM_001037663.1 | T |  |  |  |  | HSPE1 | 3336 | NM_002157.2 | T |  |  |  |
| EEF1B2 | 1933 | NM_001959.3 | T |  |  |  |  | HSPE1 | 3336 | NM_002157.2 | T |  |  |  |
| TOMM7 | 54543 | NM_019059.2 |  |  |  |  |  | P2RY5 | 10161 | NM_005767.5 |  |  |  |  |
| TOMM7 | 54543 | NM_019059.2 |  |  |  |  |  | RPS17P16 | 402057 | LOC402057 (p) | T | R |  |  |
| RPL35P1 | 440737 | LOC440737 (p) | T | R |  |  |  | RPS29P9 | 648343 | LOC648343 (p) | T | R |  |  |
| RPL35P2 | 646766 | LOC646766 (p) | T | R |  |  |  | LOC643870 | 643870 | LOC643870 (p) |  |  |  |  |
| LOC648622 | 648622 | LOC648622(d) |  |  |  |  |  | CD69 | 969 | NM_001781.2 |  |  |  | P |
| RPS27 | 6232 | NM_001030.4 | T | R |  |  |  | PHF5A | 84844 | NM_032758.3 | T |  |  |  |
| RPS15A | 6210 | NM_001030009.1 | T | R |  |  |  | SNHG5 | 387066 | NR_003038.2 | T |  |  |  |
| RPS18 | 6222 | NM_022551.2 | T | R |  |  |  | \| Key \| \| \| --- \| --- \| \| T \| Translation or protein production \| \| R \| Ribosomal \| \| E \| Energy production \| \| P \| Proliferation \| \| (p) \| pseudogene \| \| (d) \| discontinued \| | | | | | | |
| RPS15A | 6210 | NM_001019.4 | T | R |  |  |  |  |  |  |  |  |  |  |
| RPL27 | 6155 | NM_000988.3 | T | R |  |  |  |  |  |  |  |  |  |  |
| RPL31 | 6160 | NM_000993.4 | T | R |  |  |  |  |  |  |  |  |  |  |
| LOC641848 | 641848 | LOC641848 (d) |  |  |  |  |  |  |  |  |  |  |  |  |
| RPS7P3 | 728973 | LOC728973 (p) | T | R |  |  |  |  |  |  |  |  |  |  |
| RPL7P9 | 653702 | LOC653702 (p) | T | R |  |  |  |  |  |  |  |  |  |  |
