# Supplementary material for: Ribosomal and Immune Transcripts Associate with Relapse in Acquired ADAMTS13-Deficient Thrombotic Thrombocytopenic Purpura
Source: PLoS One. 2015 Feb 11;10(2):e0117614. doi: 10.1371/journal.pone.0117614 (PMC4324966; doi:10.1371/journal.pone.0117614)
Supplement: S2 Table — Gene order matches clustering in Fig. 1. (DOCX) [file pone.0117614.s002.docx]

**Table S2.**

| **Symbol** | **Entrez ID** | **Accession** | **IFN-regulated** |
| --- | --- | --- | --- |
| MT2A | 4502 | NM_005953.3 | yes |
| MT1A | 4489 | NM_005946.2 | yes |
| UBE2L6 | 9246 | NM_004223.4 | yes |
| LY6E | 4061 | NM_002346.2 | yes |
| OASL | 8638 | NM_198213.1 | yes |
| IRF7 | 3665 | NM_004029.2 | yes |
| IFITM3 | 10410 | NM_021034.2 | yes |
| IFIT3 | 3437 | NM_001031683.2 | yes |
| IFIT1 | 3434 | NM_001548.3 | yes |
| RSAD2 | 91543 | NM_080657.4 | yes |
| IFIT3 | 3437 | NM_001031683.2 | yes |
| IFIT3 | 3437 | NM_001031683.2 | yes |
| OAS3 | 4940 | NM_006187.2 | yes |
| IFI6 | 2537 | NM_022872.2 | yes |
| MX1 | 4599 | NM_0202462.3 | yes |
| HERC5 | 51191 | NM_016323.2 | yes |
| OAS1 | 4938 | NM_001032409.1 | yes |
| EPSTI1 | 94240 | NM_033255.2 | yes |
| OAS2 | 4939 | NM_016817.2 | yes |
| OAS1 | 4938 | NM_001032409.1 | yes |
| XAF1 | 54739 | NM_199139.2 | yes |
| IFI44L | 10964 | NM_006820.2 | yes |
| ISG15 | 9636 | NM_005101.3 | yes |
| OAS1 | 4938 | NM_001032409.1 | yes |
| IL1RN | 3557 | NM_173842.2 | yes |
| TMEM140 | 55281 | NM_018295.3 | no |
| CDKN1A | 1026 | NM_000389.4 | yes |
| ACTA2 | 59 | NM_001613.2 | no |
| LGALS9 | 3965 | NM_009587.2 | yes |
| TMEM158 | 25907 | NM_015444.2 | no |
| SEPT5 | 5413 | NM_002688.5 | no |
| GP9 | 2815 | NM_000174.3 | no |
| ITGB5 | 3693 | NM_002213.3 | no |
| ITGB5 | 3693 | NM_002213.3 | no |
| ACRBP | 84519 | NM_032489.2 | no |
| NRGN | 4900 | NM_006176.2 | no |
| FOXO4 | 4303 | NM_005938.3 | no |
| FKBP8 | 23770 | NM_012181.3 | no |
| ATP6V0C | 527 | XM_001130742.1 | no |
| GRINA | 2907 | NM_001009184.1 | no |
| C16orf35 | 8131 | NM_001077350.2 | no |
| SEC14L1 | 6397 | NM_003003.3 | no |
| TSC22D3 | 1831 | NM_004089.3 | no |
| IL8RB | 3579 | NM_001557.3 | yes |
| REPS2 | 9185 | NM_004726.2 | no |
| PDLIM7 | 9260 | NM_213636.1 | no |
