# Supplementary material for: Ribosomal and Immune Transcripts Associate with Relapse in Acquired ADAMTS13-Deficient Thrombotic Thrombocytopenic Purpura
Source: PLoS One. 2015 Feb 11;10(2):e0117614. doi: 10.1371/journal.pone.0117614 (PMC4324966; doi:10.1371/journal.pone.0117614)
Supplement: S3 Table — *Average expression (AVG) and standard deviation (SD) in normalized units. P-values are from the associative t-test. Functional annotations are taken from the US National Center for Biotechnology Information (NCBI) database and the literature. (DOCX) [file pone.0117614.s003.docx]

**Table S3.**

| **Key** | |
| --- | --- |
| **I** | Immune System |
| **N** | Neutrophils |
| **M** | Motility/Cytoskeleton |
| **C** | Association with Cancer |
| (p) | pseudogene |
| (d) | discontinued sequence |

| **SYMBOL** | **ACCESSION** | **NO**  **RELAPSE AVG±SD*** | **RELAPSE AVG±SD*** | **RATIO NO RELAPSE: RELAPSE** | **p-VALUE** | **FUNCTION** | | | |
| --- | --- | --- | --- | --- | --- | --- | --- | --- | --- |
| TGM3 | NM_003245.3 | 53.0±42.7 | 18.7±17.7 | 2.83 | 3.3E-07 |  |  |  |  |
| SERPINA1 | NM_001002236.2 | 44.1±26.4 | 19.3±10.8 | 2.28 | 1.3E-10 | **I** | **N** |  |  |
| REPS2 | NM_004726.2 | 21.3±13.9 | 10.5±13.9 | 2.03 | 1.2E-08 |  |  | **M** | **C** |
| KRT23 | NM_015515.3 | 106.6±58.9 | 53.8±31.3 | 1.98 | 1.3E-07 |  |  | **M** | **C** |
| HIP1 | NM_005338.5 | 26.6±20.1 | 13.5±8.1 | 1.97 | 1.5E-05 | **I** | **N** | **M** | **C** |
| LOC93432 | XM_926137.1 (p) | 24.6±14.8 | 12.7±7.7 | 1.94 | 2.8E-06 | **I** | **N** |  |  |
| MME | NM_000902.3 | 745.4±395.0 | 393.5±248.6 | 1.89 | 5.2E-06 | **I** | **N** |  | **C** |
| MGAM | NM_004668.2 | 43.8±25.0 | 23.2±12.7 | 1.89 | 2.4E-05 | **I** | **N** |  |  |
| HS.570385 | HS.570385 | 26.3±15.8 | 14.2±10.2 | 1.85 | 2.0E-06 |  |  |  |  |
| PSG3 | NM_021016.3 | 128.3±82.2 | 70.4±34.4 | 1.82 | 7.4E-07 | **I** | **N** |  |  |
| IL8 | NM_000584.3 | 81.0±58.0 | 44.5±19.0 | 1.82 | 1.4E-05 | **I** | **N** | **M** |  |
| PSG9 | NM_002784.3 | 91.7±58.8 | 50.4±24.0 | 1.82 | 1.5E-06 | **I** | **N** |  |  |
| LOC642103 | XM_936233.1 (d) | 92.1±52.0 | 50.8±27.7 | 1.81 | 3.2E-05 |  |  |  |  |
| SLC2A3P2 | XM_372780.3 (p) | 38.3±22.0 | 21.2±9.6 | 1.80 | 8.4E-08 | **I** | **N** |  |  |
| SIGLEC5 | NM_003830.3 | 78.6±42.8 | 44.0±37.9 | 1.79 | 2.2E-07 | **I** | **N** |  |  |
| MANSC1 | NM_018050.2 | 37.0±19.3 | 20.8±18.0 | 1.78 | 3.6E-08 |  |  |  |  |
| SIPA1L2 | NM_020808.3 | 116.3±61.7 | 66.0±23.8 | 1.76 | 1.0E-07 | **I** | **N** | **M** |  |
| ANPEP | NM_001150.2 | 183.3±95.5 | 104.9±58.5 | 1.75 | 4.3E-06 | **I** | **N** |  | **C** |
| TRPM6 | NM_017662.4 | 58.2±30.9 | 33.5±13.3 | 1.74 | 9.5E-07 |  |  |  |  |
| PAK2 | XM_001126110.1 | 36.8±17.1 | 21.2±7.9 | 1.74 | 4.3E-10 | **I** | **N** | **M** | **C** |
| REPS2 | NM_004726.2 | 151.4±80.9 | 87.5±72.7 | 1.73 | 8.6E-07 |  |  | **M** | **C** |
| REPS2 | NM_004726.2 | 62.0±34.2 | 35.9±32.9 | 1.73 | 1.0E-06 |  |  | **M** | **C** |
| NAMPT | NM_005746.2 | 477.6±260.0 | 276.2±220.9 | 1.73 | 8.4E-08 | **I** | **N** |  |  |
| REPS2 | NM_001080975.1 | 49.9±26.9 | 28.9±28.2 | 1.73 | 1.3E-07 |  |  | **M** | **C** |
| CR1 | NM_000651.4 | 24.5±10.8 | 14.2±7.5 | 1.72 | 7.6E-07 | **I** | **N** |  | **C** |
| ST6GALNAC2 | NM_006456.2 | 31.1±14.3 | 18.2±10.2 | 1.71 | 2.4E-09 |  |  |  |  |
| ACOX1 | NM_007292.5 | 102.3±57.8 | 59.8±34.7 | 1.71 | 6.9E-07 |  |  |  |  |
| LOC284757 | NM_001004305.1 (p) | 44.4±22.4 | 26.0±13.7 | 1.70 | 2.7E-05 |  |  |  |  |
| VNN2 | NM_004665.2 | 1221.3±611.2 | 717.7±254.2 | 1.70 | 2.6E-08 | **I** | **N** |  |  |
| NOV | NM_002514.3 | 76.8±45.7 | 45.2±35.5 | 1.70 | 2.8E-05 |  |  |  | **C** |
| TLE3 | NM_005078.2 | 31.4±17.1 | 18.5±10.3 | 1.70 | 6.7E-06 |  |  |  | **C** |
| CR1 | NM_000573.3 | 32.3±14.2 | 19.1±7.7 | 1.69 | 3.0E-07 | **I** | **N** |  | **C** |
| BTNL8 | NM_024850.2 | 23.9±12.9 | 14.2±11.7 | 1.69 | 7.1E-07 | **I** | **N** |  |  |
| PRRG4 | NM_024081.5 | 24.0±15.1 | 14.3±11.3 | 1.68 | 2.0E-06 | **I** | **N** |  |  |
| LOC642678 | XM_926130.1 (d) | 20.5±8.7 | 12.2±5.5 | 1.68 | 2.8E-05 |  |  |  |  |
| SLC26A8 | NM_052961.3 | 50.6±28.3 | 30.2±25.4 | 1.68 | 2.2E-05 |  |  |  |  |
| HS.163346 | HS.163346 | 45.7±21.5 | 27.2±13.3 | 1.68 | 3.4E-06 |  |  |  |  |
| FLJ45445 | NM_001004321.2 (d) | 27.1±13.5 | 16.1±5.6 | 1.68 | 6.1E-06 |  |  |  |  |
| PANX2 | NM_052839.3 | 187.4±86.2 | 112.0±51.0 | 1.67 | 2.5E-07 |  |  | **M** |  |
| SULF2 | NM_018837.3 | 143.5±68.0 | 85.9±29..9 | 1.67 | 3.2E-06 |  |  |  | **C** |
| CSF2RB | NM_000395.2 | 35.3±17.8 | 21.1±18.2 | 1.67 | 2.0E-09 | **I** | **N** |  |  |
| S100P | NM_005980.2 | 454.1±278.7 | 272.3±191.3 | 1.67 | 7.5E-07 | **I** | **N** |  | **C** |
| ABCG1 | NM_016818.2 | 39.6±25.5 | 23.8±10.0 | 1.66 | 3.2E-05 | **I** |  |  |  |
| FPR2 | NM_001462.3 | 338.4±171.5 | 203.9±145.5 | 1.66 | 3.8E-07 | **I** | **N** |  |  |
| PPP1R3B | NM_024607.3 | 20.2±10.8 | 12.2±6.5 | 1.65 | 2.2E-05 |  |  |  |  |
| CXCR1 | NM_000634.2 | 311.3±153.2 | 190.3±149.1 | 1.64 | 6.3E-08 | **I** | **N** | **M** |  |
| ASF1B | NM_018154.2 | 28.9±14.0 | 17.7±9.7 | 1.63 | 2.7E-06 |  |  |  | **C** |
| USP10 | NM_005153.2 | 322.1±129.3 | 197.5±97.2 | 1.63 | 1.7E-06 |  |  |  | **C** |
| LOC652616 | XM_942152.1 (p)(d) | 322.5±146.1 | 197.8±110.4 | 1.63 | 2.9E-07 | **I** | **N** |  |  |
| MANSC1 | NM_018050.2 | 64.2±30.9 | 39.6±29.1 | 1.62 | 2.5E-05 |  |  |  |  |
| LOC652578 | XM_942097.1 (d) | 23.6±13.1 | 14.6±7.3 | 1.62 | 2.6E-05 |  |  |  |  |
| WLS | NM_001002292.3 | 55.2±30.1 | 34.1±10.5 | 1.62 | 1.1E-05 |  |  |  |  |
| CNN2 | NM_004368.2 | 60.8±28.3 | 37.6±19.2 | 1.62 | 1.4E-07 | **I** | **N** | **M** |  |
| DHRS12 | NM_024705.1 | 25.4±9.2 | 15.8±4.8 | 1.61 | 4.8E-09 |  |  |  |  |
| PLXNC1 | NM_005761.2 | 60.8±30.0 | 38.0±20.6 | 1.60 | 6.9E-06 | **I** | **N** | **M** |  |
| F2RL1 | NM_005242.4 | 72.2±34.2 | 45.2±26.3 | 1.60 | 2.8E-05 | **I** | **N** | **M** |  |
| MAPK14 | NM_139012.2 | 31.7±15.7 | 19.8±15.6 | 1.60 | 1.0E-06 | **I** |  |  |  |
| LOC641710 | XM_930648.1 (d) | 29.7±14.6 | 18.6±8.3 | 1.60 | 7.1E-06 |  |  |  |  |
| VNN3 | NR_028290.1 (p) | 50.4±24.0 | 31.7±23.7 | 1.59 | 1.4E-06 | **I** | **N** |  |  |
| MAK | NM_005906.4 | 34.7±15.6 | 21.8±12.3 | 1.59 | 5.1E-06 |  |  |  | **C** |
| MCL1 | NM_021960.4 | 211.1±87.5 | 133.2±53.2 | 1.58 | 2.2E-09 | **I** | **N** |  | **C** |
| DYSF | NM_003494.3 | 411.7±207.7 | 260.0±224.4 | 1.58 | 1.6E-05 | **I** |  | **M** |  |
| Hs.131087 | Hs.131087 | 90.0±39.8 | 56.9±17.8 | 1.58 | 3.1E-07 |  |  |  |  |
| IL6R | NM_000565.3 | 53.2±26.9 | 33.7±17.1 | 1.58 | 1.2E-05 | **I** | **N** |  |  |
| CLEC4E | NM_014358.2 | 31.8±15.3 | 20.2±10.7 | 1.58 | 3.5E-06 |  |  |  |  |
| HCAR3 | NM_006018.2 | 73.0±31.5 | 46.3±31.0 | 1.58 | 3.5E-05 | **I** | **N** |  |  |
| LOC728417 | XM_001130364.3 (p) | 33.1±14.9 | 21.1±7.0 | 1.57 | 5.8E-07 |  |  |  |  |
| TSC22D3 | NM_004089.3 | 128.5±67.5 | 81.8±43.7 | 1.57 | 9.4E-06 | **I** |  |  | **C** |
| CDA | NM_001785.2 | 277.7±146.3 | 177.2±119.4 | 1.57 | 2.6E-05 | **I** | **N** |  | **C** |
| PDLIM7 | NM_213636.1 | 85.4±45.0 | 54.6±33.7 | 1.56 | 1.2E-05 |  |  | **M** |  |
| FCGR2A | NM_021642.3 | 69.6±33.8 | 44.5±22.5 | 1.56 | 2.0E-05 | **I** | **N** |  |  |
| GTF2IP1 | NR_002206.2 (p) | 75.6±37.7 | 48.4±20.3 | 1.56 | 6.4E-06 |  |  |  |  |
| NCF1B | NR_003186.1 (p) | 104.7±48.7 | 67.3±27.0 | 1.56 | 9.2E-06 | **I** | **N** |  |  |
| STARD10 | NM_006645.2 | 52.5±23.7 | 33.7±11.3 | 1.56 | 1.5E-08 |  |  |  | **C** |
| NPL | NM_030769.2 | 338.2±146.8 | 217.6±104.6 | 1.55 | 5.2E-07 |  |  |  |  |
| LIMK2 | NM_016733.2 | 47.5±23.9 | 30.6±26.4 | 1.55 | 1.1E-05 |  |  | **M** | **C** |
| STEAP4 | NM_024636.3 | 46.2±19.8 | 29.8±14.2 | 1.55 | 7.7E-07 | **I** |  |  | **C** |
| LOC728417 | XM_001130364.3 (p) | 32.2±16.0 | 20.8±8.4 | 1.55 | 1.6E-05 |  |  |  |  |
| LAMP2 | NM_013995.2 | 303.7±163.5 | 195.8±90.8 | 1.55 | 2.2E-05 | **I** | **N** |  |  |
| EMR2 | NM_152916.1 | 318.4±116.1 | 205.5±102.8 | 1.55 | 1.5E-07 | **I** | **N** | **M** | **C** |
| SLC43A2 | NM_152346.1 | 170.7±92.0 | 110.2±47.2 | 1.55 | 1.2E-05 |  |  |  |  |
| SPAG9 | NM_003971.5 | 23.2±8.3 | 15.0±4.9 | 1.55 | 1.3E-10 |  |  |  | **C** |
| SULF2 | NM_018837.3 | 194.5±81.4 | 125.8±35.5 | 1.55 | 7.0E-06 |  |  |  | **C** |
| PICALM | NM_007166.3 | 42.3±18.9 | 27.4±11.1 | 1.54 | 2.7E-07 |  |  |  |  |
| Hs.559665 | Hs.559665 | 109.1±51.0 | 70.7±30.4 | 1.54 | 7.0E-06 |  |  |  |  |
| FPR2 | NM_001005738.1 | 544.0±256.5 | 352.5±212.9 | 1.54 | 7.4E-06 | **I** | **N** |  |  |
| FOS | NM_005252.3 | 306.0±144.3 | 199.2±99.3 | 1.54 | 6.4E-06 | **I** | **N** |  |  |
| ACTN1 | NM_001102.3 | 803.3±369.2 | 525.7±271.3 | 1.53 | 2.4E-06 | **I** | **N** | **M** |  |
| RAB11FIP1 | NM_025151.4 | 105.6±50.4 | 69.3±33.2 | 1.52 | 3.1E-05 |  |  | **M** | **C** |
| UBN1 | NM_001079514.1 | 430.4±202.2 | 282.5±130.2 | 1.52 | 5.2E-06 |  |  |  |  |
| VNN3 | NR_028290.1 (p) | 163.2±66.4 | 107.2±55.7 | 1.52 | 1.2E-06 | **I** | **N** |  |  |
| F2RL1 | NM_005242.4 | 143.2±63.4 | 94.1±48.6 | 1.52 | 2.6E-05 | **I** | **N** | **M** |  |
| NCF1 | NM_000265.4 | 1119.7±440.9 | 736.0±399.6 | 1.52 | 8.3E-08 | **I** | **N** |  |  |
| WDR52 | NM_018338.3 | 48.1±20.1 | 31.6±19.0 | 1.52 | 1.8E-06 |  |  |  |  |
| KIAA0226L | NM_025113.2 | 112.6±42.5 | 74.3±28.7 | 1.52 | 2.2E-05 |  |  | **M** | **C** |
| PTPRC | NM_080923.2 | 96.6±37.5 | 63.7±24.4 | 1.51 | 5.2E-07 | **I** |  |  |  |
| DHRS12 | NM_024705.1 | 35.9±18.9 | 23.7±7.5 | 1.51 | 5.8E-06 |  |  |  |  |
| SDCBP | NM_001007067.1 | 714.0±256.4 | 474.7±197.5 | 1.50 | 4.8E-08 |  |  | **M** |  |
