# Supplementary material for: Ribosomal and Immune Transcripts Associate with Relapse in Acquired ADAMTS13-Deficient Thrombotic Thrombocytopenic Purpura
Source: PLoS One. 2015 Feb 11;10(2):e0117614. doi: 10.1371/journal.pone.0117614 (PMC4324966; doi:10.1371/journal.pone.0117614)
Supplement: S4 Table — * Average expression (AVG) and standard deviation (SD) in normalized units. P-values are from the associative t-test. Functional annotations are taken from the US National Center for Biotechnology Information (NCBI) database and the literature. (DOCX) [file pone.0117614.s004.docx]

**Table S4.**

| **Key** | |
| --- | --- |
| **T** | Translation or protein production |
| **R** | Ribosomal |
| **M** | Metabolism |
| **P** | Proliferation |
| **A** | Antigen Presentation |
| **I** | Immune |
| (p) | pseudogene |
| (d) | discontinued sequence |

| **SYMBOL** | **ACCESSION** | **RELAPSE AVG*** | **NO**  **RELAPSE AVG*** | **RATIO RELAPSE:**  **NO RELAPSE** | **p-VALUE** | **FUNCTION** | | | | | |
| --- | --- | --- | --- | --- | --- | --- | --- | --- | --- | --- | --- |
| DDX3Y | NM_004660.3 | 21.1±1.6 | 1.6±7.6 | 13.0 | 5.1E-09 | **T** |  |  | **P** |  |  |
| EIF1AY | NM_004681.2 | 74.8±123.9 | 9.9±46.2 | 7.6 | 1.7E-15 | **T** |  |  |  |  |  |
| JARID1D | NM_004653.4 | 22.8±35.3 | 3.3±15.3 | 6.9 | 3.7E-06 |  |  |  |  |  |  |
| CYorf15A | NM_001005852.2 (p) | 29.9±43.7 | 6.6±12.3 | 4.5 | 6.5E-10 |  |  |  |  |  |  |
| RPS4Y2 | NM_001039567.2 | 151.2±226.0 | 35.5±88.3 | 4.3 | 1.6E-06 | **T** | **R** |  |  |  |  |
| HLA-DRB5 | NM_002125.3 | 763.3±523.9 | 226.8±421.6 | 3.4 | 3.2E-10 |  |  |  |  | **A** | **I** |
| LOC643007 | NM_001101383.1 (p) | 280.9±216.3 | 114.3±105.7 | 2.5 | 3.9E-07 | **T** | **R** |  |  |  |  |
| RPL26 | NM_000987.3 | 640.8±534.8 | 262.4±260.5 | 2.4 | 1.2E-05 | **T** | **R** |  |  |  |  |
| LOC642250 | XM_925774.1 (p) | 35.9±25.0 | 14.9±10.8 | 2.4 | 1.9E-07 | **T** | **R** |  |  |  |  |
| LOC442454 | NR_002308.1 (p) | 362.2±297.1 | 153.3±163.5 | 2.4 | 4.1E-06 |  |  | **M** |  |  |  |
| IFNG | NM_000619.2 | 23.0±24.6 | 9.9±8.1 | 2.3 | 1.6E-09 |  |  |  |  |  | **I** |
| UQCRB | NM_006294.4 | 30.9±24.8 | 13.4±11.5 | 2.3 | 5.5E-06 |  |  | **M** |  |  |  |
| LOC402057 | NM_001080499.1 (p) | 135.3±104.0 | 59.6±48.9 | 2.3 | 9.7E-06 | **T** | **R** |  |  |  |  |
| LOC648659 | XM_944469.1 (p) | 68.7±49.5 | 31.2±27.8 | 2.2 | 1.2E-05 | **T** | **R** |  |  |  |  |
| LOC645968 | XM_928934.1 (p) | 221.6±154.4 | 101.8±87.8 | 2.2 | 6.1E-06 | **T** | **R** |  |  |  |  |
| LOC402677 | XM_943039.1 (p) | 29.4±20.5 | 13.6±12.0 | 2.2 | 8.4E-06 | **T** | **R** |  |  |  |  |
| LOC641849 | XM_935589.1 (d) | 162.1±115.9 | 76.0±65.5 | 2.1 | 1.1E-05 | **T** | **R** |  |  |  |  |
| LOC653702 | NM_001093763.1 (p) | 284.5±154.6 | 137.2±88.0 | 2.1 | 1.4E-08 | **T** | **R** |  |  |  |  |
| LOC651064 | XM_944489.1 (p) | 209.7±148.8 | 102.1±71.5 | 2.1 | 1.6E-06 |  |  |  |  |  |  |
| HLA-DRB1 | NM_002124.3 | 657.8±391.7 | 322.0±335.7 | 2.0 | 4.5E-06 |  |  |  |  | **A** | **I** |
| LOC648343 | XR_018327.1 (p) | 435.0±242.7 | 216.3±130.7 | 2.0 | 2.1E-07 | **T** | **R** |  |  |  |  |
| COMMD6 | NM_203497.2 | 512.5±349.1 | 255.9±207.5 | 2.0 | 3.2E-05 |  |  |  |  |  |  |
| CCDC72 | NM_015933.3 | 271.0±177.2 | 138.6±97.0 | 2.0 | 1.6E-06 |  |  |  |  |  |  |
| EEF1B2 | NM_001959.3 | 60.0±39.4 | 30.7±16.4 | 2.0 | 4.5E-08 | **T** |  |  |  |  |  |
| LOC641848 | XM_935588.1 (d) | 849.5±497.4 | 435.1±325.9 | 2.0 | 4.2E-06 | **T** | **R** |  |  |  |  |
| LSM3 | NM_014463.2 | 83.4±49.6 | 43.4±26.5 | 1.9 | 1.5E-06 | **T** |  |  |  |  |  |
| C14orf112 | NM_016468.6 | 46.6±27.3 | 24.4±14.9 | 1.9 | 1.1E-06 |  |  | **M** |  |  |  |
| LOC653773 | XM_938755.2 (p) | 1511.7±914.0 | 803.0±654.8 | 1.9 | 2.3E-05 | **T** | **R** |  |  |  |  |
| C14orf156 | NM_031210.4 | 184.3±104.0 | 98.0±59.5 | 1.9 | 1.5E-06 | **T** |  |  |  |  |  |
| RPS27L | NM_015920.3 | 262.3±157.3 | 139.7±96.7 | 1.9 | 9.4E-06 | **T** | **R** |  |  |  |  |
| EEF1B2 | NM_021121.3 | 56.8±33.8 | 30.3±17.8 | 1.9 | 4.6E-07 | **T** |  |  |  |  |  |
| LOC652608 | XM_942140.1 (p) | 35.5±17.3 | 19.2±9.7 | 1.9 | 2.2E-08 | **T** | **R** |  |  |  |  |
| LOC728973 | XR_015809.2 (p) | 1312.3±742.6 | 710.7±491.3 | 1.8 | 1.7E-05 | **T** | **R** |  |  |  |  |
| MRPL47 | NM_020409.2 | 37.7±18.3 | 20.4±9.6 | 1.8 | 3.5E-08 | **T** | **R** |  |  |  |  |
| LSM3 | NM_014463.2 | 105.2±59.7 | 57.4±39.7 | 1.8 | 7.8E-06 | **T** |  |  |  |  |  |
| LOC643870 | XM_927140.1 (p) | 128.0±63.0 | 70.0±39.2 | 1.8 | 7.1E-08 |  |  |  |  |  |  |
| RPL21 | NM_000982.3 | 1627.9±895.3 | 891.8±546.3 | 1.8 | 5.6E-07 | **T** | **R** |  |  |  |  |
| CETN3 | NM_004365.2 | 27.3±15.5 | 15.0±10.6 | 1.8 | 2.5E-05 |  |  |  |  |  |  |
| DBI | NM_020548.6 | 73.5±39.8 | 40.4±20.6 | 1.8 | 1.1E-06 |  |  | **M** | **P** |  |  |
| CIP29 | NM_033082.3 | 190.3±111.4 | 105.4±55.0 | 1.8 | 4.1E-07 | **T** |  |  | **P** |  |  |
| TOMM5 | NM_001001790.2 | 60.1±30.1 | 33.6±18.3 | 1.8 | 1.1E-06 |  |  |  |  |  |  |
| RPL23 | NM_000978.3 | 1845.9±984.8 | 1033.1±664.9 | 1.8 | 8.6E-06 | **T** | **R** |  |  |  |  |
| RPL7 | NM_000971.3 | 654.6±320.8 | 367.0±243.0 | 1.8 | 3.6E-06 | **T** | **R** |  |  |  |  |
| C1orf41 | NM_016126.2 | 38.8±20.9 | 21.8±11.0 | 1.8 | 7.2E-07 |  |  |  |  |  |  |
| EEF1B2 | NM_001959.3 | 922.1±507.1 | 518.1±326.5 | 1.8 | 5.1E-06 | **T** |  |  |  |  |  |
| PFDN5 | NM_002624.3 | 1372.2±744.3 | 774.7±476.0 | 1.8 | 4.4E-06 | **T** |  |  |  |  |  |
| ITGB3BP | NM_014288.4 | 22.8±9.9 | 12.9±10.2 | 1.8 | 6.4E-07 |  |  |  | **P** |  |  |
| PFDN5 | NM_145897.2 | 1295.2±680.3 | 737.2±450.8 | 1.8 | 4.7E-06 | **T** |  |  |  |  |  |
| LOC642250 | NM_001089592.1 (p) | 1709.8±874.1 | 976.4±643.4 | 1.8 | 2.2E-05 | **T** | **R** |  |  |  |  |
| HSPCAL3 | NM_001040141.1 (p) | 26.0±12.4 | 14.9±4.8 | 1.7 | 2.3E-12 | **T** |  |  |  |  |  |
| SNRPG | NM_003096.2 | 415.2±218.9 | 238.0±125.7 | 1.7 | 1.4E-06 | **T** |  |  |  |  |  |
| RPL17 | NM_001035006.2 | 2127.8±1079.8 | 1224.6±835.7 | 1.7 | 2.9E-05 | **T** | **R** |  |  |  |  |
| MRPL13 | NM_014078.5 | 56.6±30.4 | 32.6±18.1 | 1.7 | 9.7E-06 | **T** | **R** |  |  |  |  |
| CD69 | NM_001781.2 | 134.1±60.2 | 77.5±38.7 | 1.7 | 4.0E-07 |  |  |  | **P** |  | **I** |
| FAM26F | NM_001010919.1 | 188.5±78.1 | 108.9±38.2 | 1.7 | 6.4E-11 |  |  |  |  |  |  |
| ATP5I | NM_007100.3 | 31.3±16.4 | 18.1±9.3 | 1.7 | 1.5E-06 |  |  | **M** |  |  |  |
| TPRKB | NM_016058.2 | 30.2±15.7 | 17.7±8.7 | 1.7 | 1.4E-06 |  |  |  |  |  |  |
| MRPL22 | NM_014180.3 | 37.1±18.3 | 21.7±9.7 | 1.7 | 6.1E07 | **T** | **R** |  |  |  |  |
| GZMA | NM_006144.3 | 498.3±366.9 | 291.4±139.6 | 1.7 | 2.6E-05 |  |  |  |  |  | **I** |
| MRPS18C | NM_016067.2 | 111.2±53.8 | 65.5±34.3 | 1.7 | 5.9E-06 | **T** | **R** |  |  |  |  |
| RPS17 | NM_001021.3 | 841.6±441.1 | 496.6±309.7 | 1.7 | 3.1E-05 | **T** | **R** |  |  |  |  |
| C8orf59 | NM_001099672.1 | 217.1±115.0 | 128.2±65.9 | 1.7 | 5.7E-06 |  |  |  |  |  |  |
| LSM5 | NM_012322.2 | 78.8±38.4 | 46.7±18.3 | 1.7 | 2.6E-08 | **T** |  |  |  |  |  |
| PSMA4 | NM_002789.4 | 441.0±203.2 | 261.7±115.3 | 1.7 | 6.2E-08 |  |  |  |  | **A** |  |
| EEF1B2 | NM_001037663.1 | 1581.1±821.4 | 938.8±617.4 | 1.7 | 2.4E-05 | **T** |  |  |  |  |  |
| UQCRH | NM_006004.2 | 341.5±127.8 | 205.9±70.4 | 1.7 | 9.4E-12 |  |  | **M** |  |  |  |
| GEMIN6 | NM_024775.9 | 29.8±15.2 | 18.0±9.3 | 1.7 | 7.5E-06 | **T** | **R** |  |  |  |  |
| LOC642989 | XM_926370.1 (d) | 1520.2±666.9 | 918.2±510.0 | 1.7 | 3.8E-06 | **T** | **R** |  |  |  |  |
| LOC388532 | XM_939726.2 (p) | 2117.6±920.7 | 1281.4±713.3 | 1.7 | 2.2E-06 | **T** | **R** |  |  |  |  |
| MRPS28 | NM_014018.2 | 47.0±20.3 | 28.4±14.8 | 1.7 | 4.8E-06 | **T** | **R** |  |  |  |  |
| HSPE1 | NM_002157.2 | 205.8±88.4 | 124.6±54.2 | 1.7 | 1.1E-07 | **T** |  |  |  |  |  |
| MRPL3 | NM_007208.3 | 24.8±10.1 | 15.0±6.5 | 1.7 | 8.0E-07 | **T** | **R** |  |  |  |  |
| DBI | NM_001079863.1 | 330.0±161.5 | 200.3±105.6 | 1.6 | 7.8E-06 |  |  | **M** | **P** |  |  |
| NDUFS4 | NM_002495.2 | 174.2±84.6 | 106.0±55.9 | 1.6 | 4.9E-06 |  |  | **M** |  |  |  |
| LOC387934 | XM_937508.2 (p) | 28.8±14.2 | 17.6±6.8 | 1.6 | 3.8E-08 |  |  |  |  |  |  |
| RPL26L1 | NM_016093.2 | 20.2±9.5 | 12.4±6.3 | 1.6 | 1.4E-05 | **T** | **R** |  |  |  |  |
| P2RY5 | NM_005767.5 | 216.3±109.1 | 132.8±70.1 | 1.6 | 1.7E-06 |  |  |  |  |  | **I** |
| MRPL50 | NM_019051.2 | 89.9±38.5 | 55.2±31.4 | 1.6 | 9.2E-06 | **T** | **R** |  |  |  |  |
| PSMA4 | NM_002789.4 | 181.5±66.7 | 111.5±46.2 | 1.6 | 5.6E-09 |  |  |  |  | **A** |  |
| TATDN1 | NM_032026.3 | 42.2±18.1 | 26.0±11.7 | 1.6 | 9.2E-07 |  |  |  |  |  |  |
| MRPL36 | NM_032479.3 | 111.9±44.4 | 68.9±21.4 | 1.6 | 1.2E-11 | **T** | **R** |  |  |  |  |
| HSPE1 | NM_002157.2 | 198.0±90.7 | 122.0±59.0 | 1.6 | 3.3E-06 | **T** |  |  |  |  |  |
| C1orf156 | NM_033418.1 | 22.6±10.3 | 13.9±6.8 | 1.6 | 5.5E-06 |  |  |  |  |  |  |
| CRSP9 | NM_004270.4 | 22.5±10.5 | 13.9±7.2 | 1.6 | 8.8E-06 | **T** |  |  |  |  |  |
| NDUFS5 | NM_004552.2 | 259.5±106.4 | 160.4±67.4 | 1.6 | 6.8E-08 |  |  | **M** |  |  |  |
| KLRB1 | NM_002258.2 | 548.8±253.0 | 339.2±127.0 | 1.6 | 1.7E-07 |  |  |  |  |  | **I** |
| ZNF181 | NM_001029997.3 | 28.4±11.2 | 17.6±9.9 | 1.6 | 3.5E-06 |  |  |  |  |  |  |
| RWDD1 | NM_001007464.1 | 327.0±144.0 | 202.4±87.6 | 1.6 | 5.6E-07 |  |  |  |  |  |  |
| EEF1E1 | NM_004280.4 | 85.9±36.4 | 53.2±28.0 | 1.6 | 1.2E-05 | **T** |  |  |  |  |  |
| PSMC6 | NM_002806.3 | 83.9±35.1 | 52.1±23.7 | 1.6 | 1.3E-07 |  |  |  |  | **A** |  |
| LOC731640 | XM_001133089.1 (p) | 2089.0±816.9 | 1297.7±666.7 | 1.6 | 1.0E-06 | **T** | **R** |  |  |  |  |
| RSL24D1 | NM_016304.2 | 473.2±220.4 | 294.2±164.6 | 1.6 | 3.0E-05 | **T** | **R** |  |  |  |  |
| CD244 | NM_016382.3 | 28.9±13.9 | 18.0±5.8 | 1.6 | 1.8E-11 |  |  |  |  |  | **I** |
| NDUFAF2 | NM_174889.4 | 39.3±18.2 | 24.5±9.8 | 1.6 | 5.0E-07 |  |  | **M** |  |  |  |
| MRPL1 | NM_020236.3 | 50.8±23.4 | 31.8±16.6 | 1.6 | 1.9E-05 | **T** | **R** |  |  |  |  |
| NUDCD2 | NM_145266.4 | 53.9±20.1 | 33.7±14.0 | 1.6 | 7.0E-08 |  |  |  |  |  |  |
| ANAPC10 | NM_014885.4 | 36.0±18.0 | 22.6±11.5 | 1.6 | 2.4E-05 |  |  |  | **P** |  |  |
| SEC11C | NM_033280.2 | 159.1±77.8 | 100.1±44.6 | 1.6 | 3.5E-06 | **T** |  |  |  |  |  |
| ELOVL6 | NM_024090.2 | 28.3±19.4 | 17.8±7.7 | 1.6 | 1.0E-05 |  |  |  |  |  |  |
| MRPL22 | NM_001014990.2 | 116.1±51.3 | 73.2±27.6 | 1.6 | 1.6E-07 | **T** | **R** |  |  |  |  |
| CD52 | NM_001803.2 | 1242.1±515.7 | 783.5±306.0 | 1.6 | 3.9E-07 |  |  |  |  |  | **I** |
| MAF | NM_001031804.2 | 32.9±13.8 | 20.7±8.5 | 1.6 | 1.8E-08 |  |  |  |  |  | **I** |
| PPIG | NM_004792.2 | 31.1±15.2 | 19.7±9.4 | 1.6 | 9.5E-06 | **T** |  |  |  |  |  |
| DCTN6 | NM_006571.3 | 24.9±10.1 | 15.8±5.0 | 1.6 | 1.5E-09 |  |  |  |  |  |  |
| HOPX | NM_139212.3 | 357.1±223.3 | 226.0±87.0 | 1.6 | 3.3E-06 |  |  |  |  |  | **I** |
| LOC402644 | XM_938297.1 (p) | 440.4±170.2 | 278.8±88.4 | 1.6 | 1.2E-09 |  |  |  |  |  | **I** |
| COX7C | NM_001867.2 | 1027.0±435.9 | 651.7±375.5 | 1.6 | 1.8E-06 |  |  | **M** |  |  |  |
| LOC648000 | XM_371757.5 (p) | 2204.0±885.0 | 1399.9±825.4 | 1.6 | 5.5E-07 | **T** | **R** |  |  |  |  |
| NUDCD2 | NM_145266.4 | 44.2±18.0 | 28.2±10.1 | 1.6 | 8.7E-08 |  |  |  |  |  |  |
| LOC401397 | XM_001132317.1 | 117.9±45.6 | 75.1±32.0 | 1.6 | 4.9E-07 |  |  |  |  |  |  |
| UQCRH | NM_006004.2 | 551.9±211.9 | 352.0±138.0 | 1.6 | 1.8E-07 |  |  | **M** |  |  |  |
| LOC650276 | XM_939363.1 (p) | 3332.7±1203.7 | 2126.7±1189.4 | 1.6 | 7.9E-07 | **T** | **R** |  |  |  |  |
| SNRPD2 | NM_004597.5 | 1201.7±488.1 | 767.4±320.0 | 1.6 | 8.2E-07 | **T** |  |  |  |  |  |
| NDC80 | NM_006101.2 | 20.3±9.1 | 13.0±5.9 | 1.6 | 4.5E-06 |  |  |  | **P** |  |  |
| COMMD8 | NM_017845.3 | 58.2±25.2 | 37.2±17.4 | 1.6 | 8.3E-06 |  |  |  |  |  |  |
| C16orf61 | NM_020188.3 | 244.4±95.0 | 156.9±51.2 | 1.6 | 2.0E-09 |  |  |  |  |  |  |
| LOC346950 | XM_294473.2 (p) | 204.8±112.0 | 131.9±49.7 | 1.6 | 2.0E-05 | **T** | **R** |  |  |  |  |
| PSMA3 | NM_002788.3 | 73.9±31.3 | 47.6±17.7 | 1.6 | 1.5E-07 |  |  |  |  | **A** |  |
| LSM5 | NM_012322.2 | 203.1±86.0 | 131.1±55.8 | 1.5 | 8.7E-06 | **T** |  |  |  |  |  |
| UQCRHL | NM_001089591.1 | 1068.6±322.9 | 695.8±245.1 | 1.5 | 3.9E-09 |  |  | **M** |  |  |  |
| APIP | NM_015957.2 | 36.9±14.7 | 24.1±10.0 | 1.5 | 3.4E-07 |  |  |  |  |  |  |
| LOC402057 | NM_001080499.1 (p) | 3531.7±1432.8 | 2302.0±1301.9 | 1.5 | 2.1E-05 | **T** | **R** |  |  |  |  |
| ENY2 | NM_020189.5 | 144.5±70.0 | 94.2±41.4 | 1.5 | 1.4E-05 | **T** |  |  |  |  |  |
| MRPS33 | NM_016071.3 | 93.2±34.8 | 60.7±27.2 | 1.5 | 2.5E-06 | **T** | **R** |  |  |  |  |
| TAF9 | NM_016283.4 | 51.2±21.8 | 33.4±13.6 | 1.5 | 4.2E-06 | **T** |  |  |  |  |  |
| TAF9 | NM_001015891.1 | 43.6±19.0 | 28.5±9.4 | 1.5 | 1.9E-07 | **T** |  |  |  |  |  |
| MRPL32 | NM_031903.2 | 128.6±51.4 | 84.1±33.1 | 1.5 | 6.2E-07 | **T** | **R** |  |  |  |  |
| RPS17 | NM_001021.3 | 3366.9±1380.4 | 2203.5±1284.3 | 1.5 | 1.6E-05 | **T** | **R** |  |  |  |  |
| ACTR6 | NM_022496.3 | 138.0±49.7 | 90.4±40.3 | 1.5 | 1.9E-06 |  |  |  |  |  |  |
| LOC388621 | XM_941195.2 (p) | 3103.5±1027.7 | 2034.3±959.8 | 1.5 | 7.8E-07 | **T** | **R** |  |  |  |  |
| LOC646483 | XM_930551.1 (p) | 1565.5±527.9 | 1026.9±370.2 | 1.5 | 2.5E-08 | **T** | **R** |  |  |  |  |
| LOC646900 | XM_929862.1 (d) | 47.5±19.6 | 31.1±12.2 | 1.5 | 2.8E-07 |  |  |  |  |  |  |
| UQCRQ | NM_014402.4 | 355.0±131.4 | 234.0±93.6 | 1.5 | 3.5E-06 |  |  | **M** |  |  |  |
| METTL5 | NM_014168.2 | 100.9±31.3 | 66.7±27.3 | 1.5 | 1.3E-07 |  |  |  |  |  |  |
| LOC646766 | XR_017605.2 (p) | 1017.4±416.3 | 673.2±292.2 | 1.5 | 1.5E-05 | **T** | **R** |  |  |  |  |
| HINT1 | NM_005340.5 | 885.5±337.2 | 586.2±298.2 | 1.5 | 7.7E-06 |  |  |  |  |  |  |
| ACAT1 | NM_000019.3 | 125.3±48.4 | 83.0±28.4 | 1.5 | 9.8E-08 |  |  | **M** |  |  |  |
| LOC649214 | XM_945231.1 (d) | 35.4±13.8 | 23.5±8.3 | 1.5 | 1.3E-07 |  |  |  |  |  |  |
| RPS18 | NM_022551.2 | 2295.5±888.6 | 1522.3±706.4 | 1.5 | 2.2E-05 | **T** | **R** |  |  |  |  |
| NSMCE2 | NM_173685.2 | 21.0±8.8 | 14.0±5.5 | 1.5 | 2.5E-06 |  |  |  |  |  |  |
| NDUFA4 | NM_002489.3 | 787.3±285.5 | 523.3±224.4 | 1.5 | 4.8E-06 |  |  | **M** |  |  |  |
| TMEM126B | NM_018480.4 | 143.8±63.5 | 95.7±40.3 | 1.5 | 1.5E-05 |  |  |  |  |  |  |
