# Supplementary material for: Ribosomal and Immune Transcripts Associate with Relapse in Acquired ADAMTS13-Deficient Thrombotic Thrombocytopenic Purpura
Source: PLoS One. 2015 Feb 11;10(2):e0117614. doi: 10.1371/journal.pone.0117614 (PMC4324966; doi:10.1371/journal.pone.0117614)
Supplement: S5 Table — (DOCX) [file pone.0117614.s005.docx]

**Table S5.**

| Transcript | r-value^a^ | Lower 95% CI | Upper 95% CI | p-value |
| --- | --- | --- | --- | --- |
| CD52 | -0.022 | -0.348 | 0.309 | 0.898 |
| CD244 | -0.056 | -0.378 | 0.277 | 0.737 |
| GZMA | -0.037 | -0.361 | 0.295 | 0.826 |
| *HOPX* | ***-0.367*** | ***-0.621*** | ***-0.044*** | ***0.023*** |
| IFNG | -0.118 | -0.430 | 0.219 | 0.481 |
| KLRB1 | -0.208 | -0.503 | 0.129 | 0.209 |
| *MAF* | ***-0.377*** | ***-0.628*** | ***-0.055*** | ***0.020*** |
| P2RY5 | 0.068 | -0.267 | 0.388 | 0.686 |
| Ribosomal Avg | -0.032 | -0.357 | 0.299 | 0.847 |
| ^a^ Spearman correlation, 2-tailed test. | | | | |
