# Supplementary material for: Ribosomal and Immune Transcripts Associate with Relapse in Acquired ADAMTS13-Deficient Thrombotic Thrombocytopenic Purpura
Source: PLoS One. 2015 Feb 11;10(2):e0117614. doi: 10.1371/journal.pone.0117614 (PMC4324966; doi:10.1371/journal.pone.0117614)
Supplement: S6 Table — (DOCX) [file pone.0117614.s006.docx]

**Table S6.**

| Comparison | N | Median | Range | p-value^a^ |
| --- | --- | --- | --- | --- |
| Relapse | 16 | 44.5 | 9-100 | 0.233 |
| No relapse | 22 | 89 | 5-100 |  |
|  |  |  |  |  |
| RGS^b^ | 10 | 53 | 9-100 | 0.515 |
| No RGS | 28 | 83.5 | 5-100 |  |
|  |  |  |  |  |
| IFN GS^c^ | 9 | 100 | 9-100 | 0.141 |
| No IFN GS | 29 | 58 | 5-100 |  |
|  |  |  |  |  |
| HLA-DRB1^hi^ | 23 | 58 | 5-100 | 0.923 |
| HLA-DRB1^lo^ | 15 | 82 | 5-100 |  |
|  |  |  |  |  |
| HLA-DRB5^hi^ | 17 | 51 | 5-100 | 0.742 |
| HLA-DRB5^lo^ | 21 | 85 | 5-100 |  |
| ^a^ Mann-Witney U test, 2-tailed test; ^b^ RGS=ribosomal gene signature; ^c^ IFN GS= IFN gene signature. | | | | |
