# Supplementary material for: Ribosomal and Immune Transcripts Associate with Relapse in Acquired ADAMTS13-Deficient Thrombotic Thrombocytopenic Purpura
Source: PLoS One. 2015 Feb 11;10(2):e0117614. doi: 10.1371/journal.pone.0117614 (PMC4324966; doi:10.1371/journal.pone.0117614)
Supplement: S2 Fig — RBC = red blood cells, PLT = platelets, WBC = white blood cells, GRAN = granulocytes, LYMPH = lymphocytes, LYMPH = lymphocytes, MONO = monocytes, EOS = eosinophils, BASOS = basophils. (DOCX) [file pone.0117614.s008.docx]

**

**

**Figure S2.**
