# Supplementary figures and images for: Ribosomal and Immune Transcripts Associate with Relapse in Acquired ADAMTS13-Deficient Thrombotic Thrombocytopenic Purpura
Source: PLoS One. 2015 Feb 11;10(2):e0117614. doi: 10.1371/journal.pone.0117614 (PMC4324966; doi:10.1371/journal.pone.0117614)

**
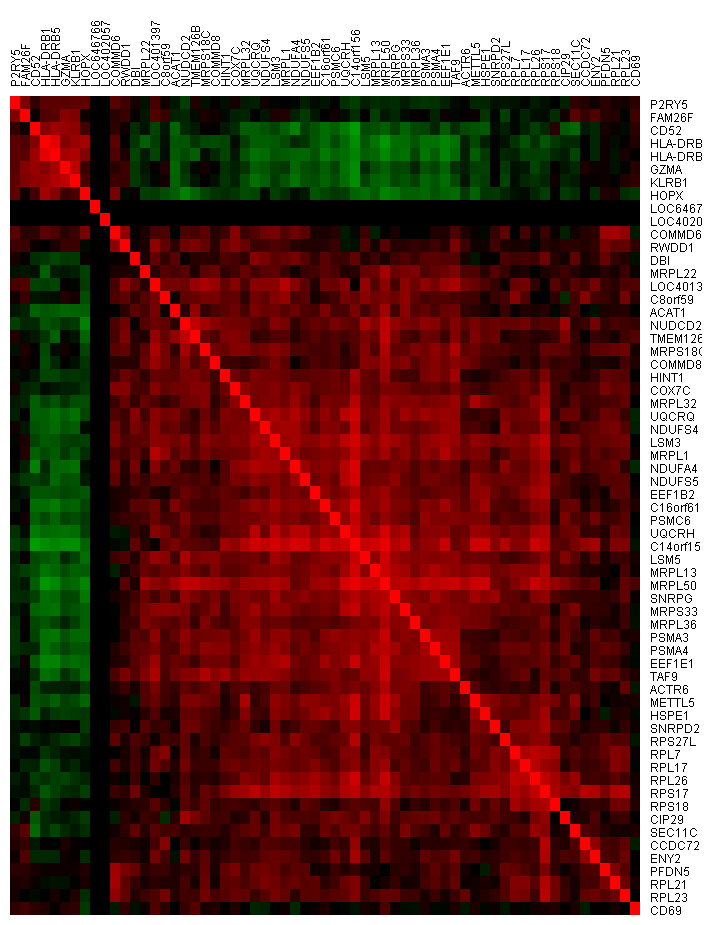
**

**Figure S3.**

Supplement: S3 Fig — Heatmap showing correlations among 63 selected genes in combined data from 3,600 non-TTP microarray datasets. Genes were selected based on their upregulation in the TTP relapse group in the present study. Red = positive Pearson’s correlation coefficient; Green = negative Pearson’s correleation coefficient. The self-self correlations (values = 1.0) can be seen on the diagonal as a reference for the relative intensities. Two groups of genes generally correlated in their expression are observed: ribosome-related genes (bottom right) and immune-related genes (top left). These two groups of genes—all up-regulated in these experiments—are not normally positively correlated. In contrast, these two groups of genes are positively correlated in TTP patients in remission. (DOCX) [file pone.0117614.s009.docx]

**
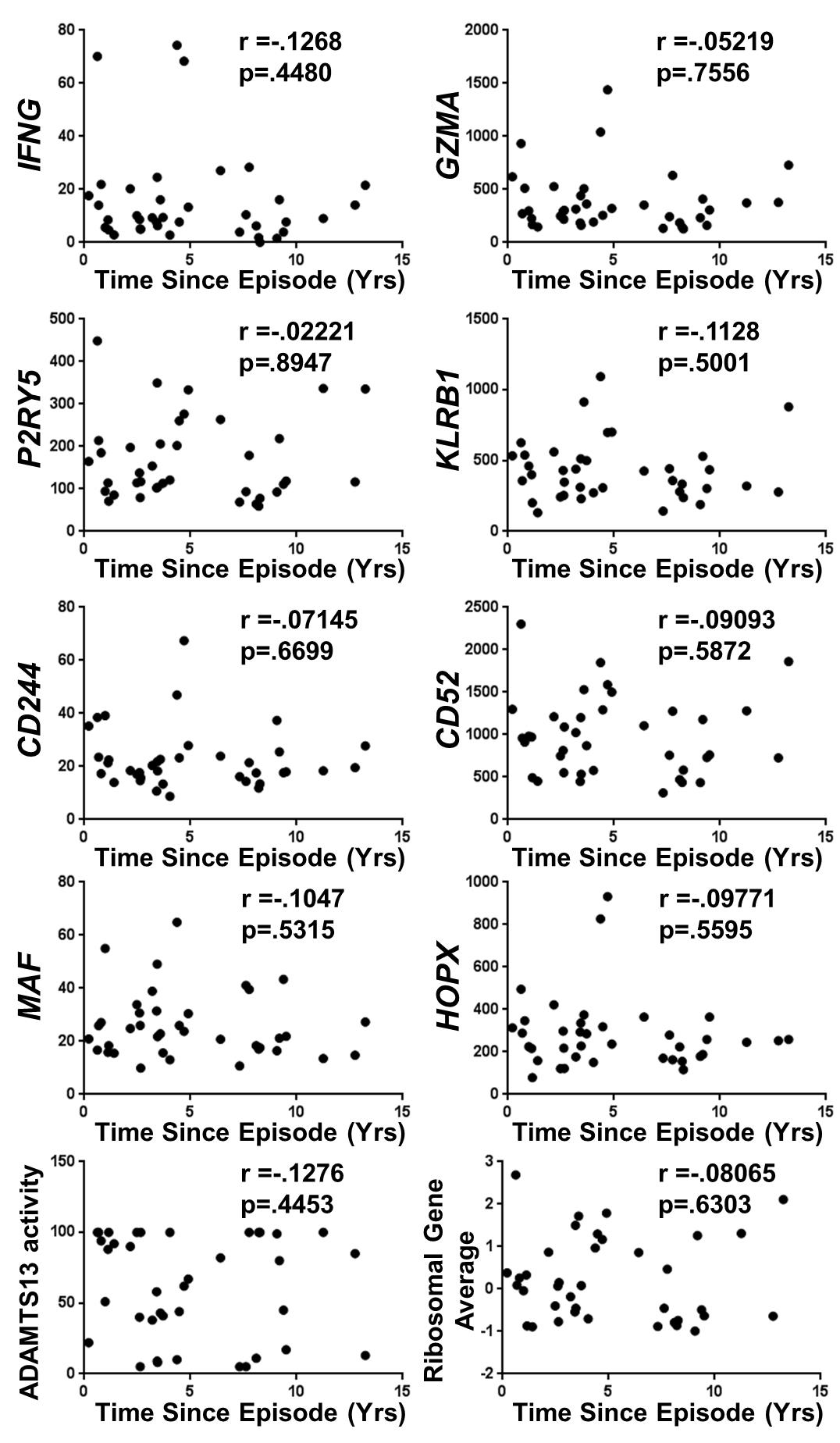
**

**Figure S6.**

Supplement: S6 Fig — For the immune genes, the Y-axis is in normalized units. ADAMTS13 activity is expressed as a percentage of maximum. Ribosomal Gene Average is expressed as average normalized expression. R values are Spearman correlations. (DOCX) [file pone.0117614.s012.docx]
