# Supplementary material for: Ribosomal and Immune Transcripts Associate with Relapse in Acquired ADAMTS13-Deficient Thrombotic Thrombocytopenic Purpura
Source: PLoS One. 2015 Feb 11;10(2):e0117614. doi: 10.1371/journal.pone.0117614 (PMC4324966; doi:10.1371/journal.pone.0117614)
Supplement: S4 Fig — Proportions were compared using Fisher’s exact test. Odds ratios (OR) were calculated using the maximum likelihood estimator (MLE). Y-axis is in normalized units. Expression levels >400 normalized units were considered high. (DOCX) [file pone.0117614.s010.docx]

**

**

**Figure S4.**
